# Identification and characterization of two putative novel genera of arteriviruses in shrews and rats

**DOI:** 10.3389/fmicb.2025.1551155

**Published:** 2025-08-04

**Authors:** Xiu Wang, Huanyu Gong, Ruixu Chen, Sumei Tan, Jianwei Shao, Jiming Chen, Shousheng Lu, Ming Liao

**Affiliations:** ^1^School of Animal Science and Technology, Foshan University, Foshan, China; ^2^Guangdong Center for Animal Disease Prevention and Control, Guangzhou, China; ^3^College of Animal Science and Technology, Zhongkai University of Agriculture and Engineering, Guangzhou, China

**Keywords:** arterivirus, shrew, rat, genome, glycosylation, mutation, evolution

## Abstract

**Introduction:**

Certain arteriviruses causing severe diseases in domestic animals, such as porcine respiratory and reproductive syndrome viruses, could originate through viral spillover, and simian arteriviruses pose threats to public health through viral spillover. To prepare for the spillover risks, it is desirable to delve into the diversity, evolution, and potential pathogenicity of arteriviruses in non-human primates, shrews, and rats, which are close in phylogenetics to humans or share the same habitats with humans and domestic animals.

**Methods:**

In this study, a variety of viruses in Asian house shrews (*Suncus murinus*) and brown rats (*Rattus norvegicus*) were detected using high-throughput sequencing and virome analysis.

**Results:**

Two viruses were identified as novel genera in the family *Arteriviridae* according to the demarcation criterion of the International Committee on Taxonomy of Viruses. The two novel arteriviruses contained arterivirus-typical genomic structures, and they were different from classified arteriviruses in the N-linked glycosylation sites of their glycoproteins. Frameshift mutations, rather than genomic recombination, played a crucial role in the genetic divergence of the two viruses.

**Discussion:**

These results expand the knowledge about the genetic diversity and evolution of shrew viruses, rodent viruses, and arteriviruses. They provide scientific data for controlling the risks posed by shrew viruses and rodent viruses to humans and livestock.

## 1 Introduction

Arteriviruses, such as porcine respiratory and reproductive syndrome viruses (PRRSVs) and equine arteritis viruses (EAVs), are spherical, variable-shaped, and enveloped viruses, 50 to 74 nm in diameter, with a linear, positive-sense, and single-stranded RNA genome, approximately 12.7–15.7 kb in length. Arteriviruses are assigned to the order *Nidovirales*, family *Arteriviridae,* which currently covers 6 subfamilies, 13 genera, 23 species, and some unclassified viruses (Brinton et al., 2021).

Multiple arteriviruses cause severe diseases in animals. For example, the two major genotypes of PRRSVs, PRRSV-1 and PRRSV-2, correspond to two species of arteriviruses: *Betaarterivirus europensis* and *Betaarterivirus americense*. Pigs of all ages are susceptible to infection with PRRSVs. Low-virulent PRRSV-1 and PRRSV-2 strains have resulted in widespread mild infections in pigs, whereas highly virulent strains of the viruses have caused severe epidemics in pigs, such as reproductive failure in sows and highly fatal respiratory illness in growing swine (Zhang et al., 2023). Most EAV infections are subclinical, but some virulent strains periodically cause significant outbreaks in horses and can be associated with abortion, neonatal mortality, and the establishment of persistent infection in stallions (Otzdorff et al., 2021). The mouse arterivirus of lactate dehydrogenase-elevating virus (LDV) causes lifelong asymptomatic and persistent infections in mice that can only be recognized by elevated levels of plasma lactate dehydrogenase (LDH). Some LDVs are neuropathogenic to mice. The arterivirus of wobbly possum disease virus (WPDV) was found in brushtail possums in New Zealand and Australia. Possums infected with WPDV have an uncoordinated “wobbly” gait (Gulyaeva et al., 2017). An arterivirus found in European hedgehogs was associated with fatal encephalitis (Dastjerdi et al., 2021). Arteriviruses were also detected in Chinese softshell turtles that had hemorrhagic disease (Liu et al., 2015).

It is believed that the two species of PRRSV, PRRSV-1 and PRRSV-2, directly originated from rat arteriviruses following spillover incidents (Wu et al., 2018). Furthermore, simian arteriviruses, particularly simian hemorrhagic fever virus (SHFV) (Warren et al., 2022), which are endemic among African primates and cause fatal hemorrhagic fevers in primates, may cause severe diseases in humans through spillover events (Bailey et al., 2016), thereby posing a considerable risk to public health (Brinton et al., 2021).

Rodent arteriviruses pertain to two subfamilies (Var*iarterivirinae* and *Heroarterivirinae*), four genera, and seven species, while shrew arteriviruses pertain to one subfamily (*Crocarterivirinae*), one genus, and three species (Brinton et al., 2021). In this study, we identified and characterized two novel genera of arteriviruses in Asian house shrews (*Suncus murinus*) and brown rats (*Rattus norvegicus*) using the high-throughput sequencing (HTS) method. Asian house shrews and brown rats are common small wild mammals sharing the same habitats with humans and domestic animals (Chen Y. M. et al., 2023; da Paz et al., 2023; Gong et al., 2024; Vanmechelen et al., 2021; Wang et al., 2017).

## 2 Materials and methods

### 2.1 Sample collection

Frozen carcasses of Asian house shrews and brown rats with unknown health status were bought were bought from March to August 2023 from a professional capturer who captured shrews and rats in various cities in Guangdong Province, China for farmers or companies, and we did not set any criteria for selecting shrews or brown rats in terms of health status, age, or sex. Asian house shrews and brown rats were identified through their morphological features. Brown rats are characterized by their brownish-gray fur, long tails, and large ears. Their tails are shorter than their bodies (excluding tails). Asian house shrews resemble small rats, with slender bodies, long pointed snouts, and large, sensitive whiskers. They have short legs and long, scaly tails. The brown rats and Asian house shrews used in this study were also confirmed with the HTS data obtained in this study, which contained various gene sequences of brown rats or Asian house shrews rather than other mammals. Their fur is soft and ranges in color from light brown to grayish, often with a white underside. The carcasses were disinfected with 75% ethanol, and their lungs, livers, and spleens were taken out for RNA purification.

### 2.2 RNA extraction

Approximately 50 mg of lung, spleen, and/or liver spleen tissue of each animal was homogenized with 500 μL lysis solution in a 2-mL centrifuge tube using two steel beads and a tissue homogenizer (TissueLyser 85,300, Qiagen) at 30 Hz and 4°C. The tissue total RNA was extracted using TRIzol LS reagent (Invitrogen) and subsequently further purified using RNeasy Plus Mini Kit (Qiagen), according to the manufacturer’s instructions. The extracted RNA was evaluated using a NanoDrop 2000 (Thermo Fisher Scientific). Subsequently, the extracted RNA solutions from certain homogenates were pooled in equal quantity, and the quality of the pooled RNA solutions was evaluated using an Agilent 2,100 Bioanalyzer (Agilent Technologies).

### 2.3 HTS and data analysis

RNA library preparation for HTS was conducted following the methods described previously (Zhang et al., 2019; Zhang et al., 2024). Briefly, ribosomal RNA (rRNA) was depleted using a Ribo-Zero-Gold (Epidemiology) kit (Illumina, San Diego, CA, USA) following the manufacturer’s instructions. The rest of the RNA was fragmented, reverse-transcribed, adapted, and purified using a TruSeq total RNA library preparation kit (Illumina). Library quality was examined by the Qubit high-sensitivity RNA/DNA assays (Thermo-Fisher, Shanghai, China) and Agilent 2,100 Bioanalyzer (Agilent, Santa Clara, CA, USA). Paired-end (150-bp) sequencing was performed on the Illumina Hiseq 2,500 platform. The library preparation and HTS were performed by Novogene (Tianjin, China).

Quality control of HTS data was performed using the FASTP software (version 0.22.0) to remove low-quality reads, adapter sequences, barcode sequences, and sequences with poor quality at the ends of the raw reads, resulting in clean reads (Chen et al., 2018). The quality of the clean reads was analyzed using the FastQC software (version 0.12.0) (de Sena Brandine and Smith, 2021). The clean reads were assembled into contigs using the Megahit software (version 1.2.9) (Li D. et al., 2015) with the default k-mer value, and the minimal contig length was set as 30 nt. The contigs were compared with the non-redundant protein database (NCBI nr database) using the Diamond software (version 2.1.8) to identify homologous sequences in the database (Buchfink et al., 2021), and the E-value was set as 0.1. The resultant daa files were visualized using the MEGAN software (version 6.24.25) for taxonomic annotation (Huson et al., 2016). The viral contigs identified were aligned using online BLAST to verify the viral annotation information and calculate sequence similarity.

### 2.4 Genomic sequencing and analysis

Genomic sequencing gaps between contigs were filled by RT-PCR and Sanger sequencing, and the genome termini of the virus were determined using 5′/3’ RACE kits (Takara, Dalian, China) as described previously (Li C. X. et al., 2015), using the primers listed in Table 1. The GenBank accession numbers of the genomic sequences of two arteriviruses reported in this study were PP947442 and PP964217.

**Table 1:** Primers for amplifying unmapped genomic sequences of a shrew arterivirus.

Targeted site	Length	Primer name	Sequence
5,014–5,557	543 bp	AHSAV-A-F	TGACGGCATCTTTGCACAAG
		AHSAV-A-R	GTGGTGGAGGTGTTGGTAAC
6,270–6,838	568 bp	AHSAV-B-F	CGGATTTGGATGAAATAGCC
		AHSAV-B-R	TTCTTCGGTCAAATGCTTGG
9,618–10,185	567 bp	AHSAV-C-F	ATGTACCTGTGCCATTGGAA
		AHSAV-C-R	TCACACAACGGTTGAGTTGT
10,482–12,026	1,544 bp	AHSAV-D-F	CTTATTGCCAAGGGCATCAT
		AHSAV-D-R	CCACAACAATGAGACTAAGC
5′-terminal	unknown	5’RACE RT1	GGGTCGAAAAATGGCCAAT
		5’RACE RT2	TGATGGTTGTGCCTTGAATGG
		5’RACE PCR1	CGGGCAATGGGGTATATGG
		5’RACE PCR2	ATAGATCGTCCGGATCCCAAT
		5’RACEouter	GCTGTCAACGATACGCTACGTAAC
		5’RACEadaptor	GCTGTCAACGATACGCTACGTAACG
			GCATGACAGTGGGGGGGGGGGGG

Contigs and reads were mapped to certain reference sequences using the Geneious software (Kearse et al., 2012), to identify genomic sequencing gaps, sequencing depth, and putative ORFs. N-glycosylation sites were predicted using NetNGlyc 1.0 (Gupta and Brunak, 2002). Transmembrane helices in proteins were predicted using the DeepTMHMM software (Hallgren et al., 2022). To evaluate the potential roles of frameshift mutations (FSMs) and genomic recombination in the divergence of arteriviruses, the relevant sequences were aligned using the stringent parameters (gap opening penalty = 3.0 and gap extension pendalty = 1.0) and the stringent E-INS-i mode in MAFFT (Katoh et al., 2019). Then, FSMs, which were constituted by the insertion or deletion of 3n ± 1 nucleotides within open reading frames (ORFs), were calculated using in-house Python scripts (Supplementary Text S1) and verified manually, and genomic recombination events were calculated using the RDP4 software (Martin et al., 2015).

### 2.5 Phylogenetic analysis

The amino acid sequences of five structural domains (3CLpro, NiRAN, RdRp, ZBD, and HEL1) in the genomes of arteriviruses were aligned using the E-INS-i mode in MAFFT (Katoh et al., 2019). Then, the best phylogenetic tree model, which was the one with the lowest BIC score, was identified using the ModelFinder tool in the PhyloSuite package (Kalyaanamoorthy et al., 2017; Zhang et al., 2020). Finally, the genetic lineage relationships were analyzed using the IQ-TREE program (Minh et al., 2020), according to the best phylogenetic tree model and the maximum likelihood method. Bootstrap support values were calculated with 1,000 replicates. At least one sequence was selected from each classified species of *Arteriviridae* for this analysis, and several sequences of PRRSV-1, PRRSV-2, and unclassified arteriviruses were selected to exhibit the diversity of arterivirid species and unclassified arteriviruses.

### 2.6 Detection of newly identified arteriviruses

To tentatively investigate the prevalence of certain arteriviruses, the lung tissue samples of 32 Asian house shrews and 200 brown rats were detected through RT-PCR, using the one-step All-Ready RT-PCR kit (Biotephy, Qingdao, China) and the primers listed in Table 2, which were located at conserved genomic regions of the relevant viruses. The RT-PCR started with reverse transcription at 50°C for 30 min followed by 15 min at 95°C and 40 cycles of amplification: 30 s at 94°C, 30 s at 55°C, 30 s at 72°C, and a final 10-min extension step at 72°C.

**Table 2:** Primers for detection of two arteriviruses.

Targeted virus	Targeted sites	Length	Primer name	Primer sequence
ARTV/shrew/FS	13,001–13,422	421 nt	ARTVshrewF	ACCAGTTGGAGCGGTGGT
			ARTVshrewR	CGTCAAGTCAACTGAGAAATCC
ARTV/rat/GD	6,937–7,560	624 nt	ARTVratF	GATGAAGCGGTGAAGATTGT
			ARTVratR	GATTCAAGACCAGACGTTCCA

### 2.7 Data calculation

Differences in sequences or glycosylation sites were calculated by the count of different sites divided by the count of total and non-redundant sites. The 95% confidence interval (95%_CI) of the prevalence was calculated using the equation 95%_CI = p ± 1.96 × (P(1-P)/*n*)^0.5, where P is the detected prevalence and *n* is the number of samples (Mendenhall et al., 2019).

## 3 Results

### 3.1 Virome detection of shrew samples

The virome of 16 (11 males weighing 42.6±5.7 g and 5 males weighing 37.3±4.6 g) Asian house shrews was detected using the HTS with the six pooled samples. The 16 shrews were randomly divided into two groups, and the total RNA of the lungs, livers, or spleens of the same group was pooled into one sample. The HTS of these six pooled samples generated 227,160,852 raw reads and 217,943,872 clean reads with high sequencing quality (Q20≥95.9%, Q30 > 91.0, and 60.5%≥(G + C)%≥50.4%), as shown in Supplementary Table S1.

**Figure 1:**
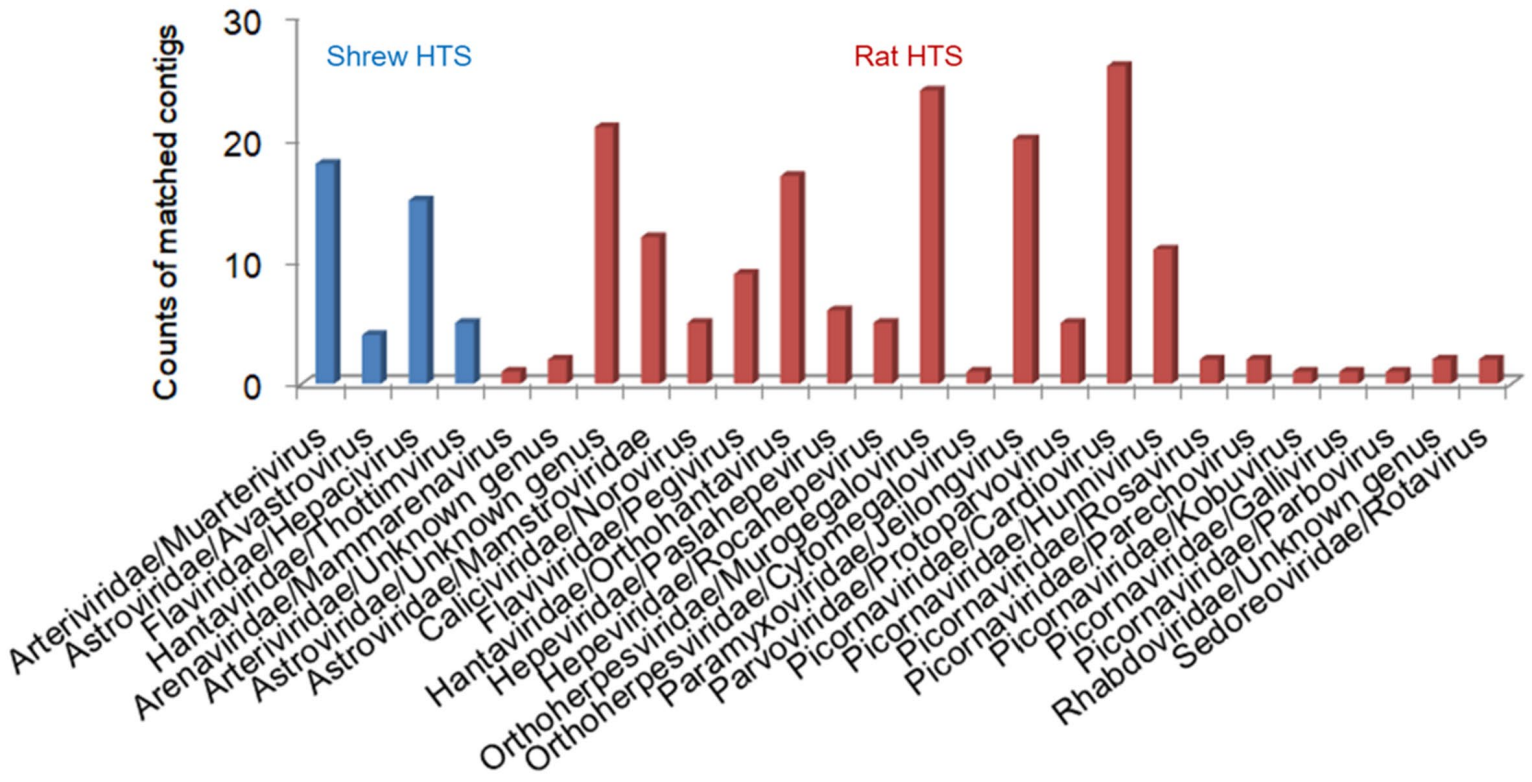
The animal viruses detected through high-throughput sequencing (HTS) from the samples of shrews (blue) and brown rats (red).

From the clean reads, 235,657 contigs were assembled. Among the contigs, 42 assembled from 14,383 clean reads belonged to four vertebrate animal viruses in the families of *Arteriviridae*, *Astroviridae*, *Flaviridae*, and *Hantaviridae* (Figure 1). These viruses were further confirmed through the online BLAST analysis at NCBI. The arterivirus was identified in both lung pooled samples and both spleen pooled samples, but not in the liver samples. The astrovirus and the hantavirus were both identified in only one lung pooled sample, and the flavivirus was identified in all six pooled samples. These four viruses were all identified in the lung pooled samples (Table 3).

**Table 3:** Detection of four viruses in six pooled tissue samples of Asian house shrews*

	Lung 1	Lung 2	Liver 1	Liver 2	Spleen 1	Spleen 2
Flavivirus	+	+	+	+	+	+
Arterivirus	+	+	−	−	+	+
Astrovirus	+	−	−	−	−	−
Hantavirus	−	+	−	−	−	−

* +: positive; −: negative.

The online BLAST of the relevant contigs suggested that the shrew astrovirus was the most homologous to the unclassified virus Wenzhou rodent astrovirus 1 in *Astroviridae.* The shrew flavivirus was the most homologous to the unclassified virus Wenzhou *Suncus murinus* hepacivirus 1 in the genus *Hepacivirus* of *Astroviridae.* The shrew hantavirus was the most homologous to *Thottimvirus thottapalayamense* in the genus *Thottimvirus* of *Hantaviridae.* The shrew arterivirus, which was termed the Foshan strain of Asian house shrew arterivirus (ARTV/shrew/FS), was the most homologous to the Gkd-1 strain of Olivier’s shrew virus 1 (OSV-1/Gkd) in the genus *Muarterivirus* of *Arteriviridae*, but their sequence identities were only 35.4–72.5% (Vanmechelen et al., 2018).

### 3.2 Viromic detection of brown rat samples

The virome of 100 brown rats was detected using the HTS. The total RNA of the lung and spleen tissue of each rat was extracted and pooled into six HTS samples, and each HTS sample corresponded to the extracted RNA of 15–20 rats. The six samples generated 253,602,536 raw reads and 243,122,710 clean reads with high sequencing quality (Q20 ≥ 97.5%, Q30 ≥ 93.5%, and (G + C)% ≥ 50.6% and ≤54.2%), as shown in Supplementary Table S1.

From the clean reads, 223,507 contigs were assembled. Among the contigs, 175 assembled from 58,493 clean reads belonged to 24 species of vertebrate animal viruses in 13 families (Figure 1), including zoonotic norovirus in *Caliciviridae*, Seoul virus in *Hantaviridae*, pegivirus in *Flaviviridae*, hepatitis E virus in *Hepeviridae*, Kobuvirus in *Picornaviridae*, and rotavirus B in *Sedoreoviridae*, besides some viruses in *Arenaviridae, Arteriviridae*, *Astroviridae*, *Orthoherpesviridae*, *Paramyxyoviridae*, *Parvoviridae,* and *Rhabdoviridae*. The rat arterivirus, which was termed the Guangdong strain of rat arterivirus (ARTV/rat/GD), was more homologous to LDV than to other classified viruses, but their genomic sequence identity was only 61.9%.

### 3.3 Genomic analysis of two novel arteriviruses

**Figure 2:**
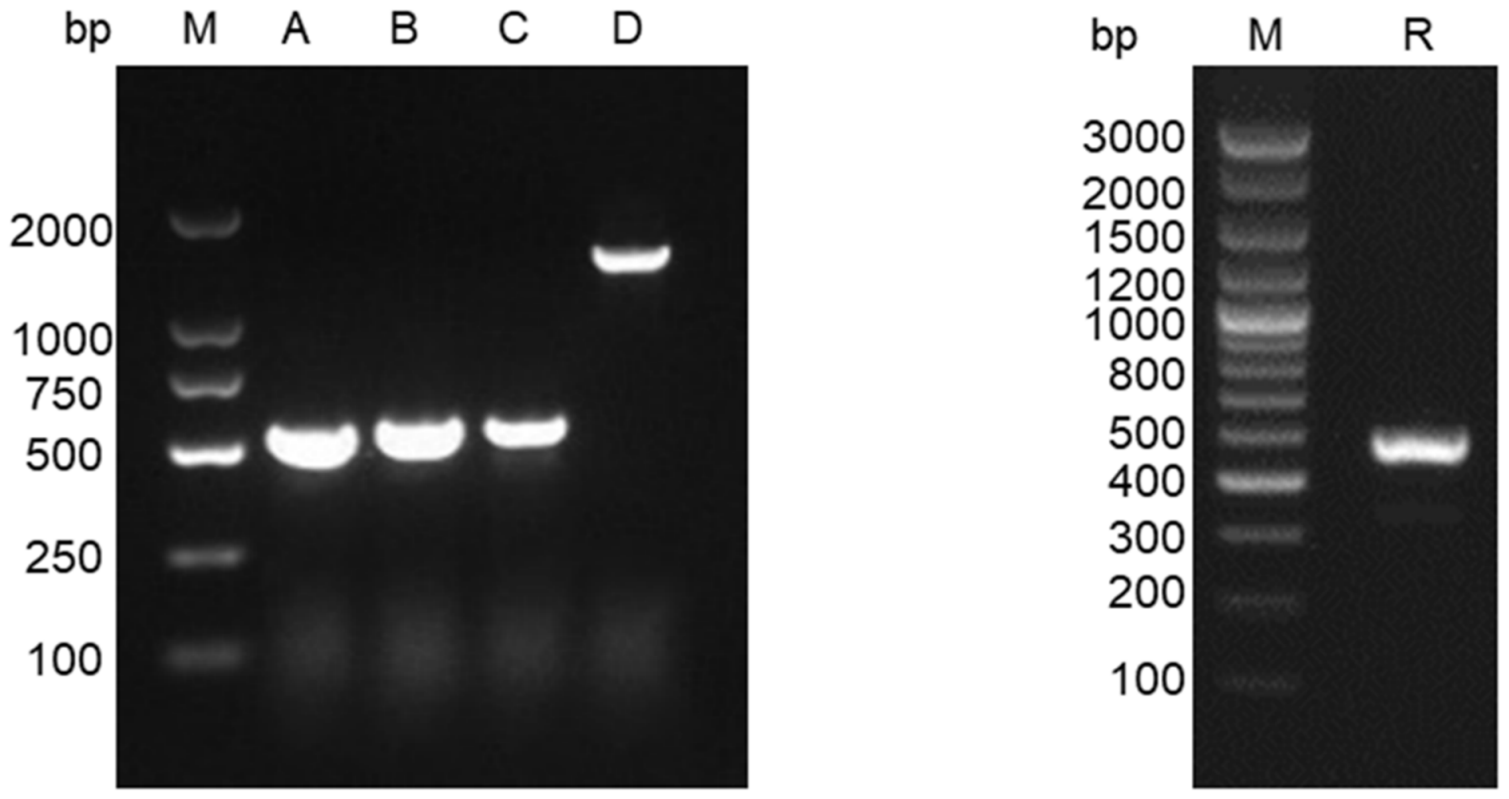
Electrophoresis of the RT-PCR amplicons of ARTV/shrew/FS for Sanger sequencing. Lane M: DNA marker; Lanes A, B, C, and D: the amplicons using the primer pairs from top down given in Table 1. Lane R: the amplicons of 5’-RACE for sequencing the 5′-terminal of the genome of ARTV/shrew/FS.

The entire genomic sequence of ARTV/rat/GD was obtained directly through HTS. The entire genomic sequence of ARTV/shrew/FS was obtained through the HTS and Sanger sequencing of the products of RT-PCR and 5’-RACE (Figure 2).

The two genomes were 14,184 nt and 13,619 nt in length. Except for a few sites at the 5′ termini, each site of the two genomes was sequenced by the HTS by 11.4 and 37.3 times on average, as shown by the mapping of the genomes using the relevant HTS clean reads. The mapping also showed that the genomic sequences of ARTV/shrew/FS obtained through the HTS were fully consistent with the genomic sequences obtained through the Sanger sequencing.

**Figure 3:**
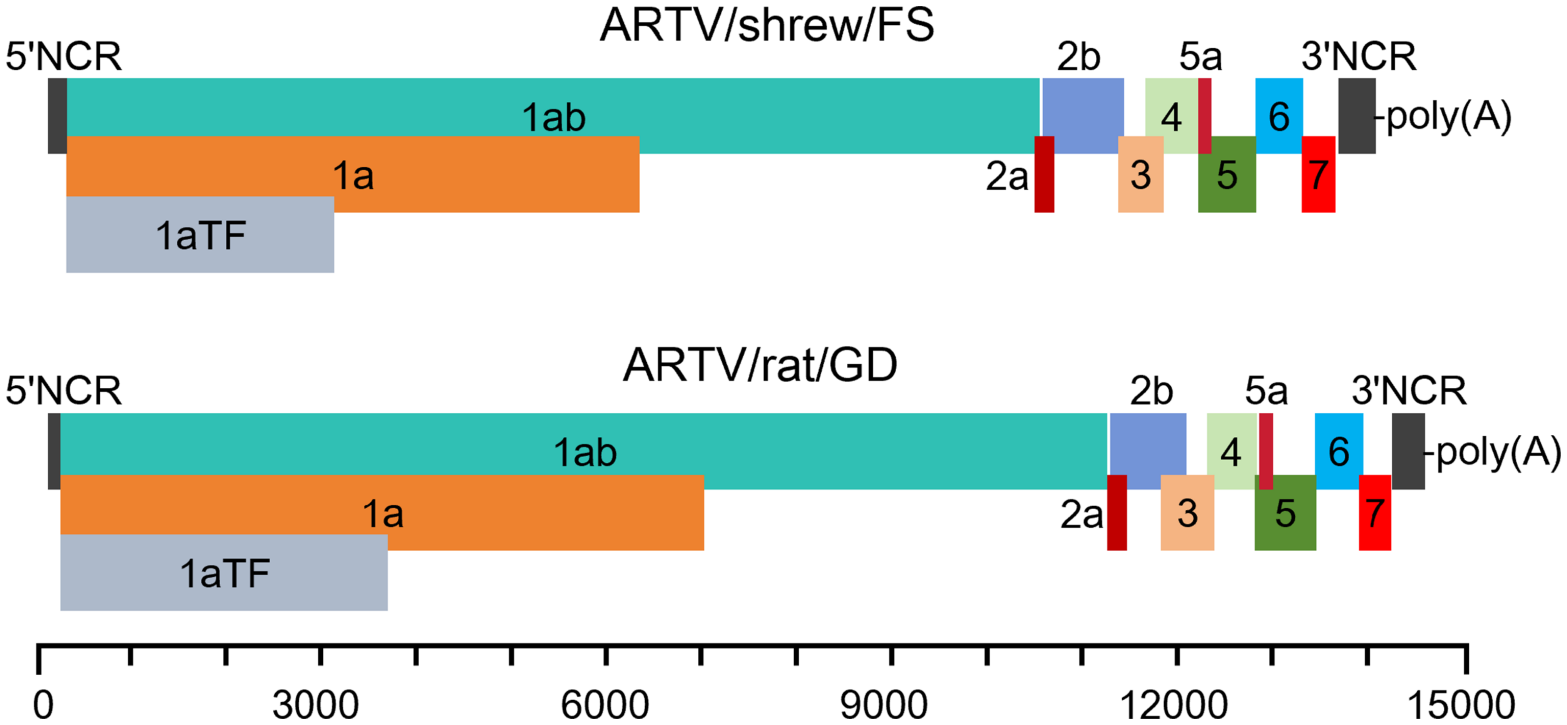
The putative genomic structures of two arteriviruses flanked by two non-coding regions (NCRs). The open reading frames (ORFs) encode the proteins of pp1aTF, ppla, pplab, GP2a(E), GP2b, GP3, GP4, GP5, GP5a, M, and N, respectively.

The genomes of two novel arteriviruses contained at least 11 arterivirus-typical ORFs: 1ab, 1a, 1aTF, 2a, 2b, 3, 4, 5, 5a, 6, and 7 that encode the viral (poly)proteins pp1ab, pp1a, pp1aTF, GP2a(E), GP2b, GP3, GP4, GP5, GP5a, M, and N, respectively (Figure 3).

The polyprotein pp1ab in *Arteriviridae* was putatively expressed via a − 1 programmed ribosomal frameshift at the end of ORF 1a. The frameshift of the two viruses occurred at the last nucleotide of the motif TTTAAAC near the end of ORF1a. The motif complies with the frameshift-stimulatory sequence in the form X_XXY_YYZ, where XXX represents any trinucleotide sequence, YYY is AAA or UUU, and Z is an A, T, or C, located a few nucleotides upstream of a stable RNA secondary structure (Li D. et al., 2015).

**Figure 4:**
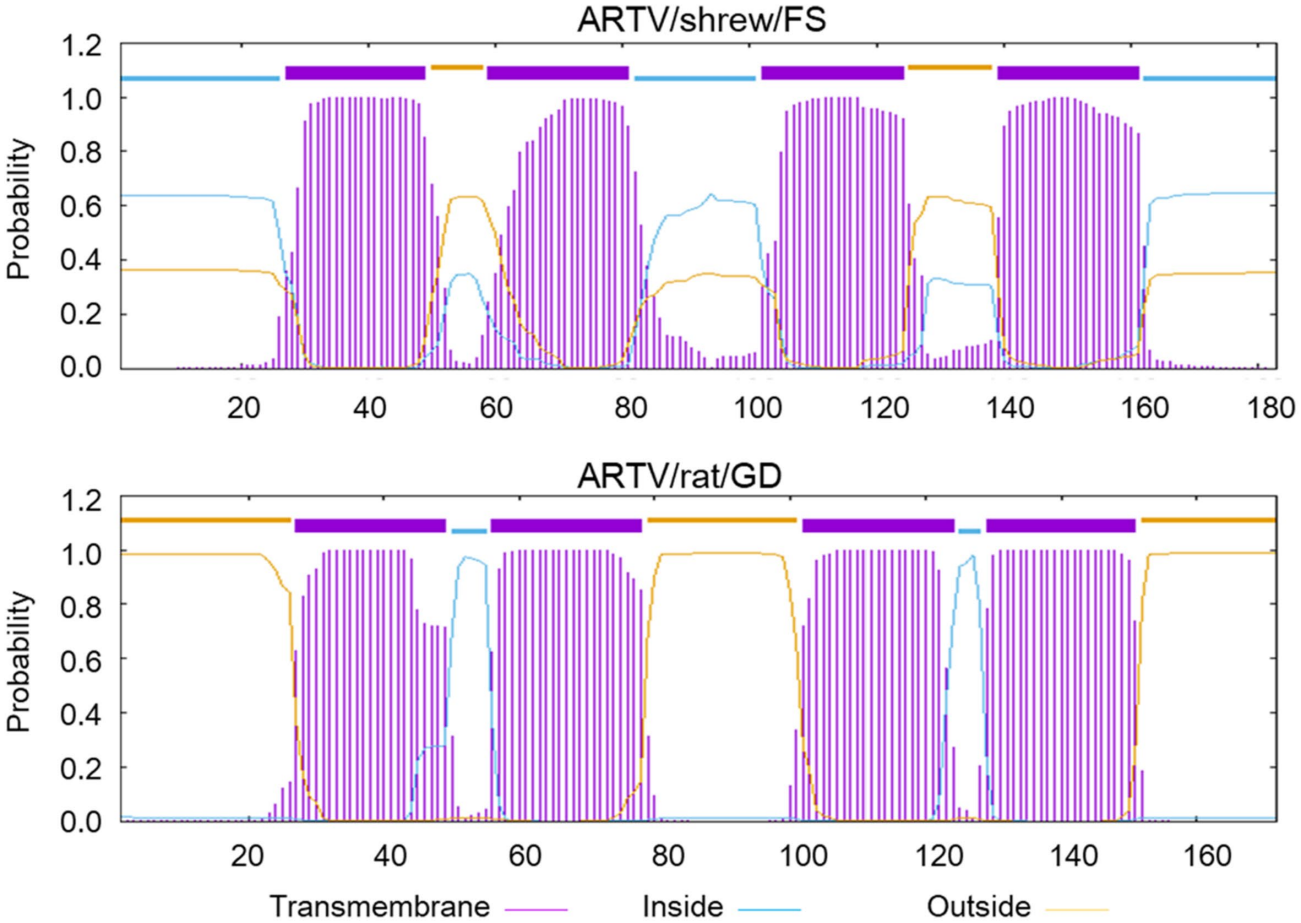
The predicted transmembrane regions within the polyprotein pp1aTF of ARTV/shrew/FS and ARTV/rat/GD.

Diverse arteriviruses employ a − 2 frameshift in the nonstructural protein 2 (nsp2) region of the 1a ORF and encode the polyprotein pp1aTF, which joins the first part of the 1a ORF with a transmembrane region encoded on a different ORF (Figure 4) (Li et al., 2019). The −2 frameshift putatively occurred at a conserved GGUUUU motif in the ORF1a of the two novel arteriviruses. In the genome of ARTV/shrew/FS, the frameshift site was located at nt 2,433–2,438, followed by the FSE (CCCCGCACC) 10 nucleotides downstream. The frameshift site in the genome of ARTV/rat/GD was located at nt 3,058–3,063, followed by the frameshift stimulatory motif (CCCATCUCC) 11 nucleotides downstream (Li D. et al., 2015).

The 5′-proximal two large ORFs of the genomes of ARTV/shrew/FS and ARTV/rat/GD, ORF1a and ORF1ab, encode the polyproteins of ppla and pplab, which are cleaved into certain nonstructural proteins (NSPs), such as nsp1α, nsp1β, nsp2, and nsp9. These NSPs mediate the viral genome RNA replication and the production of subgenomic mRNAs (Li D. et al., 2015). The 3′-proximal ORFs encode certain structural proteins of the viruses, such as E, GP2, GP3, GP4, GP5, M, and N (Figure 3).

**Figure 5:**
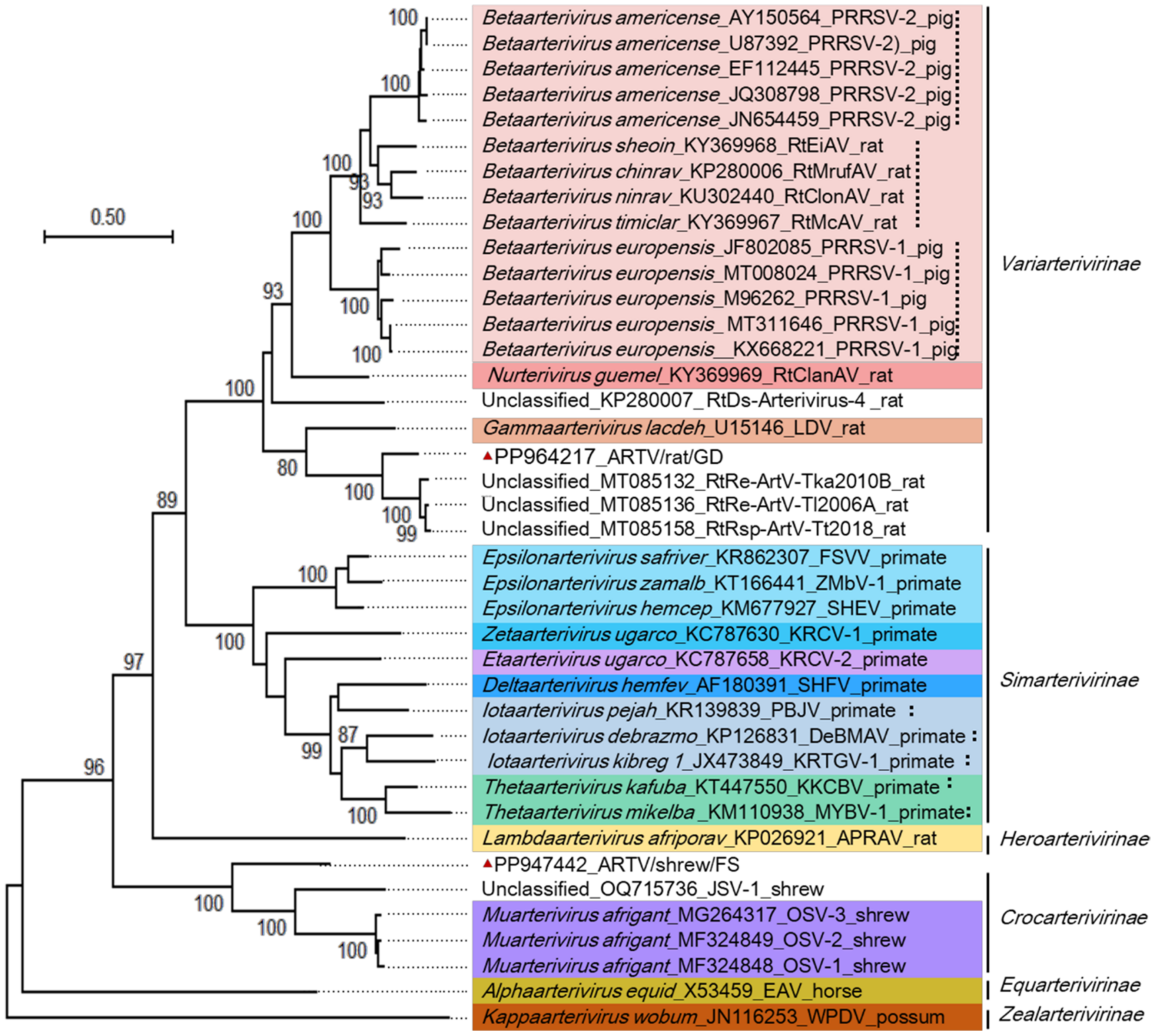
Phylogenetic relationships among certain arteriviruses. Sequences were termed with the virus species name, GenBank accession numbers, virus strain information, and host names. Different virus genera were marked with different hues. The genomic sequences reported by this study were marked with red triangles.

The N-linked glycosylation sites on the membrane glycoproteins GP2-GP5 are conserved in the same genus or species of arteriviruses but distinct between genera or subfamilies of arteriviruses. ARTV/shrew/FS and three classified shrew arteriviruses and one unclassified shrew arterivirus belonged to *Crocarterivirinae* (Figure 5), and ARTV/shrew/FS exhibited significant differences from the four other shrew arteriviruses in the N-linked glycosylation sites of the viral glycoproteins GP2-GP5 (Table 4). For instance, more than 60% of N-linked glycosylation sites in GP2a were different between ARTV/shrew/FS and four other shrew arteriviruses (Table 4). Similarly, ARTV/rat/GD and 16 other arteriviruses in 8 species and 3 genera belonged to Var*iarterivirinae* (Figure 5), and ARTV/rat/GD exhibited significant differences from these 16 arteriviruses in the N-linked glycosylation sites of the viral glycoproteins GP2-GP5. (e.g., ≥5/9 in GP3) (Table 4). For instance, more than 55% of N-linked glycosylation sites in GP2a were different between ARTV/shrew/FS and four other shrew arteriviruses (Table 4).

**Table 4:** The N-linked glycosylation sites in the glycoproteins GP2 − GP5 of some arteriviruses in two subfamilies*

Virus species_GAN_host^‡^	GP2a	GP3	GP4	GP5
*Betaarterivirus americense_*AY150564_pig	[178, 184]	29, 42, 50, 131, 152, 160, 195	37, 84, 120,130	30, 33, 44, 51
*Betaarterivirus americense_*U87392_pig	[178, 184]	29, 42, 50, 131,152, 160, 195	37, 84, 120, 130	30, 33, 44, 51
*Betaarterivirus americense_*EF112445_pig	[178, 184]	29, 42, 50, 131, 152, 160, 195	37, 84, 120, 130	30, 34, 35, 44, 51
*Betaarterivirus americense_*JQ308798_pig	[178, 184]	29, 42, 50, 70, 131,152, 160, 195	37, 84, 120, 130	34, 44, 51
*Betaarterivirus americense_*JN654459_pig	[178, 184]	29, 42, 50, 100, 131, 152, 160, 195	37, 57, 84, 120, 130	34, 44, 51
*Betaarterivirus europensis_*JF802085_pig	173, 179	29, 42, 50, 130, 151, 159, 194	37, 89, 125, 135	37, 46, 53
*Betaarterivirus europensis*_KX668221_pig	173, 179	29, 42, 50, 130, 151, 159, 194	37, 89, 125, 135	37, 46, 53
*Betaarterivirus europensis*_MT008024_pig	173, 179	29, 42, 50, 130, 151, 159, 194	37, 87, 123, 133	46, 53
*Betaarterivirus europensis*_M96262_pig	173, 179	27, 42, 50, 130, 151, 159, 194	37, 88, 124, 134	46, 53
*Betaarterivirus europensis*_MT311646_pig	173, 179	27, 42, 50, 130, 151, 159, 194	37, 88, 124, 134	37, 46, 53
*Betaarterivirus sheoin*_KY369968_rat	[169, 174, 180]	29, 42, 50, 130, 151, 159, 194	37, 58, 88, 124, 134, 149	36, 47, 54
*Betaarterivirus chinrav*_KP280006_rat	[173, 179]	27, 42, 50, 130,151, 159	37, 89, 125,135	27, 37, 44
*Betaarterivirus ninrav*_KU302440_rat	158, 164	17, 32, 40, 120, 141, 149, 184	37, 88, 124, 134	27, 37, 44
*Betaarterivirus timiclar*_KY369967_rat	133, 171, 177	[7, 12, 18, 108, 146, 151]	122, 156	37, 55
*Nuarterivirus guemel*_KY369969_rat	[16, 110, 171, 177]	[2, 23, 35, 82, 93, 139, 143, 153]	39, 117, 137	32, 41, 48, 109
*Gammaarterivirus lacdeh*_U15146_rat	157, 164, 175	23, 49, 119, 133, 142, 174	40, 83, 119, 129, 139	36, 45, 52, 155
PP964217_ARTV/rat/GD_rat	5, 155, 160, 233	33, 54, 70, 98, 128, 134, 145	64, 82, 115, 125, 135	[53, 61, 68]
*Muarterivirus afrigant*_MG264317_shrew	51, 170, 189, 236, 240, 248	50, 82, 117, 126, 137	32, 68, 120	[53, 54, 67, 74]
*Muarterivirus afrigant*_MF324849_shrew	51, 170, 189, 236, 240, 248	25, 50, 117, 126, 137	32, 68, 120	[15, 48, 61, 68]
*Muarterivirus afrigant*_MF324848_shrew	51, 170, 189, 236, 240, 248	50, 117, 126, 137	32, 68, 120	[5, 38, 51, 58]
Unclassified shrew arterivirus_OQ715736	153, 165, 220, 228, 235	54, 65, 69, 128, 137	[83, 91]	36, 49, 56
P947442_ARTV/shrew/FS_shrew	[168, 228, 232, 241]	33, 45, 52, 74, 123, 134	31, 41, 66, 118	[35, 49, 56]

^*^ The last five viruses belong to *Crocarterivirinae*, and the other viruses belong to *Variarterivirinae*; the sites unlikely to be glycosylated because the relevant proteins do not contain a signal peptide are shown with square brackets; the sites are numbered according to their corresponding sequences before alignment; the glycosylation sites unique in the same subfamily among the selected sequences are underlined. ^‡^ GAN: GenBank accession number.

The 1a gene was shorter by 735 nt in ARTV/shrew/FS than in ARTV/rat/GD. Totally, 60 insertions or deletions of nucleotides were observed in the 1a gene sequences after the alignment using the stringent model and parameters. Of the 60 insertions or deletions, 37 led to FSMs, inserting or deleting 3n-1 or 3n-2 nucleotides. Of the 37 FSMs, 14 resumed the original ORF if the ORF of one virus was set as the original ORF. At least 39.5% of the sites of the aligned 1a gene sequences encode amino acids not according to the original ORF because of the FSMs, as shown in Supplementary Text S2.

The genomic sequences of the arteriviruses shown in Figure 5 were aligned and analyzed using the eight methods (RDP, GENECONV, BootScan, MaxChi, Chimaera, SiScan, PhyIPro, and LARD) incorporated in the software RDP4. Consequently, 26 possible genomic recombination events in ARTV/shrew/FS or in ARTV/rat/GD were identified, but none of them were supported by at least three of the eight methods incorporated in the software, except for the one at sites 8,512–8,780 in the genome of ARTV/rat/GD, and this potential recombination event was refuted by phylogenetic analysis. Consequently, no reliable recombination events were identified from these genomic sequences.

### 3.4 Taxonomic analysis of the two novel arteriviruses

The pairwise differences in the combined sequence of the five domains of 3CLpro, NiRAN, RdRp, ZBD, and HEL1 were abbreviated as the 5DS differences below. As shown in Supplementary Table S2, the 5DS differences were ≥38.1% between ARTV/rat/GD and classified arteriviruses and ≥20.4 and <29.0% between ARTV/rat/GD and unclassified arteriviruses. Consequently, according to the genus demarcation criterion of *Arteriviridae* (the 5DS Difference>29.0–37.0%) and the subgenus demarcation criterion of *Arteriviridae* (the 5DS Difference>16.5–20.3%) stipulated by the International Committee on Taxonomy of Viruses (ICTV), ARTV/rat/GD and some unclassified arteriviruses can constitute a novel genus of *Arteriviridae*, and ARTV/rat/GD itself can represent a novel subgenus of *Arteriviridae*.

The 5DS differences between ARTV/shrew/GD and all other arteriviruses with known sequences, classified or unclassified, were ≥39.0%. Therefore, according to the demarcation criteria of the ICTV, ARTV/shrew/GD can represent by itself a novel genus of *Arteriviridae*.

### 3.5 Phylogenetic analysis of the two novel arteriviruses

According to the combined amino acid sequences of the five domains of 3CLpro, NiRAN, RdRp, ZBD, and HEL1, ARTV/rat/GD was closer in phylogenetics to eight unclassified rodent arteriviruses, such as RtRe-ArtV-Tka2010B and RtRe-ArtV-Tl2006A with GenBank accession numbers of MT085132 and MT085136, and ARTV/shew/FS was closer to the shrew arterivirus OSV-1/Gkd in the genus *Muarterivirus* than to other classified arteriviruses (Figure 5).

Figure 5 suggested that, according to the branch length of the phylogenetic tree that represents genetic distances between the viruses, ARTV/rat/GD and some unclassified rodent arteriviruses could constitute a new genus close to the genus *Gammaarterivirus* represented by LDV, and ARTV/shew/FS itself could constitute a new genus close to the genus *Muarterivirus* represented by OSV-1/Gkd. Figure 5 also suggested that the unclassified shrew arterivirus with the GenBank accession number OQ715736/JSV-1 could represent another novel genus of arteriviruses.

### 3.6 Detection of the two novel arteriviruses

Detection of the RNA of ARTV/shrew/FS in the lung tissues of 32 Asian house shrews, including the 16 shrews utilized for the above HTS, showed 4 positives. The 95% confidence interval of the prevalence of ARTV/shrew/FS in the captured Asian house shrews was 1.0% ~ 24.0%.

Detection of the RNA of ARTV/rat/GD in the lung tissues of 200 brown rats, including the 100 rats utilized for the above HTS, showed 2 positives. The 95% confidence interval of the prevalence of ARTV/rat/GD in the captured brown rats was 0.0–2.4%.

## 4 Discussion

In this study, a variety of vertebrate viruses in Asian house shrews and brown rats were identified through high-throughput sequencing and virome analysis, and two viruses could represent novel genera of arteriviruses. The two novel arteriviruses were characterized in terms of their genomic structures, taxonomy, N-linked glycosylation sites of the viral glycoproteins, and the roles of FSMs and genomic recombination in their genetic evolution.

The two novel arteriviruses were identified in the tissues of the lungs, spleens, and/or livers rather than the feces or intestines of shrews or rats. This suggests that they can infect and replicate in shrews or rats. The distinct tissue distribution of the four viruses identified in shrews (Table 3) could result from the fact that different viruses have different tissue tropisms.

The high sequencing quality of the clean reads (Supplementary Table S1) and the relatively high sequencing depth of ARTV/shrew/FS and ARTV/rat/GD (11.4 and 37.3) supported the reliability of the genomic sequences of ARTV/shrew/FS and ARTV/rat/GD reported in this study, which was also supported by the full consistency between the genomic sequences of ARTV/shrew/FS obtained through HTS and the genomic sequences obtained through the Sanger sequencing. Notably, the genomic sequences reported in this study, which were obtained through the HTS of pooled samples collected from various cities in a province, represented the consensus genomic sequences of the relevant viruses in a region rather than in a host.

This study suggested that ARTV/shrew/FS and ARTV/rat/GD can represent two putative novel genera of *Arteriviridae*, according to the demarcation criterion of ICTV. This is also supported by the topology of the phylogenetic tree (Figure 5) and the marked differences in the N-linked glycosylation sites of the viral glycoproteins. N-linked glycosylation in viral glycoproteins is essential for the infectivity and antigenicity of diverse viruses, such as influenza viruses and arteriviruses (Li D. et al., 2015; Rowland and Brandariz-Nuñez, 2024), and thus could be important for the interaction between arteriviruses and their hosts.

This study suggested that FSMs were likely important for the genetic divergence of the two viruses, which is consistent with a previous study showing that FSMs are important for the evolution of viruses above the species level (Ji et al., 2023). Although genomic recombination is frequent in the evolution within PRRSVs (Tian, 2017; Zhang et al., 2023), it was unlikely important for the evolution of arteriviruses between species or genera, as suggested by this study.

Unlike the fact that diverse coronaviruses have been identified in humans, bats, birds, and marine animals, arteriviruses have not been identified in these vertebrates, even though extensive HTS screening of animal viruses has been conducted in recent years (Brinton et al., 2021) and coronaviruses and arteriviruses share the same order of *Nidovirales*. The reasons and significance of this difference are elusive. Nevertheless, this fact suggests that humans are naïve to arterivirus infections, and so the potential spillover of arteriviruses from animals to humans could lead to severe disasters in public health.

The risk of the spillover of arteriviruses from shrews and rats to humans and domestic animals is considerable because shrews and rats share habitats with humans and domestic animals, and the spillover could occur through indirect contact and contaminated food. The spillover events of diverse zoonotic viruses, such as hantavirus, hepatitis E virus, Langya henipavirus, and severe fever with thrombocytopenia syndrome virus from rats or shrews to humans, have been documented (Gong et al., 2024; Liu et al., 2014; Wang et al., 2017; Zhang et al., 2022; Chen et al., 2022; Keita et al., 2021). The spillover risk is further supported by the fact that PRRSVs, which have caused severe epidemics in pigs in the 1980s, 1990s, and 2000s worldwide (Tian, 2017), likely originated through the spillover of rat arteriviruses to pigs in the 1980s (Wu et al., 2020). The findings of two novel genera of arteriviruses in this study are useful for humans to identify the potential spillover of arteriviruses from shrews and rats to humans or domestic animals in the future.

Shrews have been identified with compelling evidence as the reservoirs of multiple zoonotic viruses, such as Borna virus, Langya virus, and SFTSV (Gong et al., 2024; Liu et al., 2014; Pörtner et al., 2023; Zhang et al., 2022), and rats have been identified as the reservoirs of diverse other zoonotic viruses, such as Seoul virus, norovirus, and bocavirus (Chen R. X. et al., 2023; Niendorf et al., 2021; Xiong et al., 2019). This study revealed and characterized two novel putative genera of arteriviruses circulating in shrews and rats. It expanded the knowledge about the genetic diversity and evolution of arteriviruses, aiding in preparing for the risk of viral spillover of arteriviruses to humans and domestic animals.

The number of classified species in *Arteriviridae* has increased significantly since the 2014 ICTV release (Brinton et al., 2021). This study and several other studies suggest that the taxa of this family need to be further expanded (Brinton et al., 2021). Meanwhile, some reported arteriviruses, such as the ones with GenBank accession numbers of KP280007 and OQ686610 have not been formally classified as novel species or genera because their full genomic sequences were unavailable, even though they likely met the criteria to be a novel species or genus of *Arteriviridae*.

To better understand the diversity and evolution of arteriviruses in various animals and prepare for the relevant spillover risks, it is desirable to sequence the entire genomes of the newly identified arteriviruses and continue to delve into the diversity and evolution of arteriviruses in non-human primates or other wild mammals, such as rodents, shrews, and bats, which share the same habitats with humans and domestic animals. The novel genomic sequences and the phylogenetic findings reported in this study are useful for the design of species-specific, genus-specific, and family-specific RT-PCR methods for delving into the diversity and evolution of arteriviruses. Furthermore, it is valuable to investigate the impact of the differences in the N-linked glycosylation sites of the viral glycoproteins and the FSMs identified in this study on the infectivity, host tropism, and immune evasion of arteriviruses in the future. Additionally, the prevalence of the two arteriviruses should be detected with more shrews and brown rats captured in more regions.

## Data Availability

The datasets presented in this study can be found in online repositories. The names of the repository/repositories and accession number(s) can be found in the article/Supplementary material.
