# Supplementary material for: Identification and characterization of two putative novel genera of arteriviruses in shrews and rats
Source: Front Microbiol. 2025 Aug 4;16:1551155. doi: 10.3389/fmicb.2025.1551155 (PMC12358435; doi:10.3389/fmicb.2025.1551155)
Supplement: Supplementary file 1 [file Data_Sheet_1.pdf]

Supporting information

of the article titled

***Genomic characterization of two putative novel genera of arteriviruses in shrews and rats*.**

Text S1

The Python script for decoding frame-shift mutation in open-reading frames

(Pages 2–3)

Table S1

The quality data of the clean reads obtained in this study

(Page 4)

Text S2

The Python script for decoding frame-shift mutation in open-reading frames

(Pages 5–15)

Table S2

The 5DS Differences (the pairwise differences in the combined sequence of the five domains of 3CLpro, NiRAN, RdRp, ZBD, and HEL1) between the viruses representing classified and unclassified genera and species of arteriviruses

(Pages 16–50)

Text S1

The Python script for decoding frame-shift mutation in open-reading frames

```
import csv
import tabulate
def read_sequences(file_path):
    sequences = {}
    with open(file_path, 'r') as file:
        current_id = ""
        current_seq = ""
        for line in file:
            line = line.strip()
            if line.startswith('>'):
                if current_id:
                    sequences[current_id] = current_seq
                current_id = line[1:]
                current_seq = ""
            else:
                current_seq += line
        if current_id:
            sequences[current_id] = current_seq
    return sequences
def count_variations(seq1, seq2):
    variations = {}
    start_index = None
    insertions = 0
    deletions = 0
    for i in range(min(len(seq1), len(seq2))):
        if seq1[i] != seq2[i]:
            if seq1[i] == '-':
                deletions += 1
            elif seq2[i] == '-':
```

```
                insertions += 1
            if start_index is None:
                start_index = i
        else:
            if start_index is not None:
                variations[(start_index, i)] = (insertions, deletions)
                start_index = None
                insertions = 0
                deletions = 0
    if start_index is not None:
        variations[(start_index, len(seq1) - 1)] = (insertions, deletions)
    return variations
def print_variations_table(variations):
    table = []
    for start, end in variations.keys():
        insertions, deletions = variations[(start, end)]
        Affected_sites = insertions - deletions
        if Affected_sites != 0:
            table.append([start, end, Affected_sites])
    print(tabulate.tabulate(table, headers=['Start', 'End', 'Affected_sites'],
tablefmt='github'))
file_path = "C:\\Users\\Acer\\Desktop\\CoV1aD.fasta"
sequences = read_sequences(file_path)
seq1 = sequences['PdCoV1a']
seq2 = sequences['EU111742']
variations = count_variations(seq1, seq2)
print_variations_table(variations)
with open('C:\\Users\\Acer\\Desktop\\1aD.csv', 'w', newline='') as csvfile:
    csvwriter = csv.writer(csvfile)
    csvwriter.writerow(['Start', 'End', 'Affected_sites'])
    for start, end, Affected_sites in variations.items():
        csvwriter.writerow([start, end, Affected_sites])
```

Table S1

The quality data of the clean reads obtained in this study

Pooled samples	Raw reads	Clean reads	Quality data of clean reads		
			Q20	Q30	G+C rate
Shrew_lung_1	38907336	37208372	96.0%	91.0%	53.1%
Shrew_lung_2	30189876	28417432	95.9%	91.0%	60.5%
Shrew_liver_1	42035294	40325590	97.5%	93.3%	50.4%
Shrew_liver_2	39411552	38140958	96.9%	92.4%	55.2%
Shrew_spleen_1	36954116	35315130	96.8%	92.3%	57.6%
Shrew_spleen_2	39662678	38536390	97.6%	93.7%	55.4%
Rat_lung/spleen_1	45249822	43979716	97.6%	93.9%	51.3%
Rat_lung/spleen_2	41748994	40238652	97.5%	93.4%	53.5%
Rat_lung/spleen_3	41835282	39950754	97.1%	92.6%	52.4%
Rat_lung/spleen_4	41011082	39841524	97.4%	93.1%	51.1%
Rat_lung/spleen_5	41799922	39683444	97.6%	93.6%	50.6%
Rat_lung/spleen_6	41957434	39428620	97.9%	94.7%	54.2%

Text S2

Insertions and deletions of nucleotides in the aligned 1a gene sequences of ARTV/rat/GD and ARTV/shrew/FS

The sites marked with color represented frame-shift mutations, where 3n±1 nucleotides were inserted or deleted and changed the enocoded amino acids

```
ARTV/rat/GD    atggatgtacgccggtgtctctgcgccccgaacctaggggtgtttataagtgatgggata
ARTVshrew/FS   atgaatcccatggcctgttcgtgcaagcgcttggtcccggtcatttcggaggacggcgtt

ARTV/rat/GD    gcgttttgcaccaggtgcctttt--------------------tgaacggcccctcctcc
ARTVshrew/FS   atgtactgcgtcaagtgtttcatgcctcgcgaggcactgccggttgatgaccccgattac

ARTV/rat/GD    cactggacctacaaaaacctaact------gtggaactattgggctatattgcagctccc
ARTVshrew/FS   cggaaacggttcgggaaactggctctcttcgtagaccaattagagagattttgacaccg

ARTV/rat/GD    cacccagcaaaggattgagcctcggtgctctcgtgtcaccgacgtgctctcctgctgggc
ARTVshrew/FS   cgcccggattggga-----------------------------------tccggacgatc

ARTV/rat/GD    agtgttggttgagcgccatatacccgattgctagaatgacctccggaaatttcaactttc
ARTVshrew/FS   tatgctggatggaggccatataccccattgcccgctgcactagtggtaactggggtttcc

ARTV/rat/GD    tcagccggcttgaccgagtcgctcgcgaaatttatcgcgaaggaggactcacccagcgag
ARTVshrew/FS   gggatcgcctccagacagtattgaggcgcttgtacgcctcgcaaacttttgatcgagcag

ARTV/rat/GD    ttttgattaatcttcaggtttatgagcgagggtgctcctggttcccgattgttgggcctg
ARTVshrew/FS   tatgggatgacctcggatgctttgcgaaaggcatgaaggtgtatctctgcactgggcccg

ARTV/rat/GD    ttcctggcgttgcagtttatgcaaactcaatgcatgtgagtgacaggccgttcccggggg
ARTVshrew/FS   ttcctggtgtagccctatatttaatcgagggcactgttctggtcgacatttccccggag

ARTV/rat/GD    ctactcatgtactaactaacttgccactcaattccaacagatgt----------------
ARTVshrew/FS   ctcaatgggtgattaccaatcttccactattcaacgcctgctatttccctgaaaagtta
```

```
ARTV/rat/GD    --agggatggcttctgcccatttgatgcggcccatagccacgtctacagtctggaaggaa
ARTVshrew/FS   ggtgtggtaaccattatgcacagcttctgattgaaaaccaaggccgatttcattatgata

ARTV/rat/GD    agacaattttcattgatgggaactcatacatgtggtctcatggcactgcagcgcagtcaa
ARTVshrew/FS   accaaattttcaccattcaaggcacaaccat-----------------------------

ARTV/rat/GD    ggaagccgtggggtgaaacgctcaagctgtgcagacggattgtacgcagcttgccacctg
ARTVshrew/FS   ------------------------------------------------------cactca

ARTV/rat/GD    atcatgccatcaaaacagacttgtctggccattctgggttttcgttcaactacggactcc
ARTVshrew/FS   cctgggcggcgaaaacaaacaaattggccatttttcgacccctttgccaactaaaattcg

ARTV/rat/GD    actccggacacgtgagaatgatgcagcgtgaaggccctgattatggactctgctgggctc
ARTVshrew/FS   tgatcttgcccggaaagttcgaggagcgtggtgg--------------------------

ARTV/rat/GD    agcttttctctgattacaacacctcgtgtcgtgaggtcaatctggccgagaggtttgggt
ARTVshrew/FS   ---------gaaaagacaccttttcctgccgagtggtcagaccggc--------------

ARTV/rat/GD    accagttgcccactggggtggcgggcgcctacctcatgcggcgtctgcaggtgaacggac
ARTVshrew/FS   --------------------------------------------ttactggcgacggact

ARTV/rat/GD    tgaagttggtaatcaaggaggcagggaacactttcaccgagtgggccctaggatctgagg
ARTVshrew/FS   tagtttcggcaaaggaggctatggaataaggtttgaccatggcctctgctggatgtcaat

ARTV/rat/GD    actccta-cattagacacatcagtttgaccggtgacccggtcgaagactttttttgtcaaa
ARTVshrew/FS   gtttggagcttttggcccctgcatcgcatcccaggcgatgaataaaggatttttgactaa

ARTV/rat/GD    gtctgtgaattctccgttatggacaacactgtcaggacgaacgcgcatccataccgactg
ARTVshrew/FS   atttggaa----------------------------------------------------

ARTV/rat/GD    gccagaaaccacaggtacggatacagtcctcccggtgatggggcatgtggccttcattgc
ARTVshrew/FS   --------------------ttgaaggccccctgctgaataagtacatgtttgattatgg
```

```
ARTV/rat/GD     tggagtgctgtcatcaacaacatgaatggcggaaagctcaggactaccagcccatccgtt
ARTVshrew/FS    cttgaagttggtttacacattgaagtcggaag----------------------------

ARTV/rat/GD     accagagattccagccagtggcttgacaactttgacctcgttgccttaatccagtcccgg
ARTVshrew/FS    ------------------------------------------------------------

ARTV/rat/GD     catttgcccgtgtccctcgggaattgccctacagcatgctaccacataacgaacactggg
ARTVshrew/FS    -------ctgaatacataattagttccccagaaaaaggtcattggcagttgcacatggca

ARTV/rat/GD     gctcattgggaagtccgctgtgaccccgtcgtaggaaactggggttgcacattgactgc
ARTVshrew/FS    gtccctgagtctgaacaccttccccacgttgttgcgtatgtcaagtgcgtccccaacttt

ARTV/rat/GD     gcaatgggtgtgtgcgatggggactgcaagcctgtgcctacaatacctgtt---------
ARTVshrew/FS    ggagatacagatgaaggtcgccagtttcgatttgcacaccacgtacgtgttggctccacc

ARTV/rat/GD     ------ttagacagcattgatcatgcttttcttcactcagttgatgacctgccgtacgta
ARTVshrew/FS    caaacctcaaaccaaaagaagcgtgcccgcgctaagccagtcactcacgaccc-------

ARTV/rat/GD     gtgtcccctgatagcgttttaccacgctggacctactcacggtctttacttgcagaggat
ARTVshrew/FS    ------------------------------------------------------------

ARTV/rat/GD     gacgtgagctcgcaaatcgcggctcttggccaacagtattgtgacagccttaacatgccc
ARTVshrew/FS    -------acccgctgatggcggttgtgggcttaacgtgatttatcgcgcttggcagatag

ARTV/rat/GD     caagggatcagatccgacatgcttgactcagttgtcggcactcaaaccagtcgatcggca
ARTVshrew/FS    cgatgggtgccgagccacttgcttggggtgatttcccgt---------------------

ARTV/rat/GD     gttcgatctatgcttgagtcgcttgcggccagcaggcctcaaaaaatagtgccacctcgg
ARTVshrew/FS    ----------------------------------------------tagatcaaagtcag

ARTV/rat/GD     cacgaatcagagagtgacttgacggctttcggt-----------------------cttg
ARTVshrew/FS    tggttgtgtgaggatgatctggtgtttctaaatcaaaccttgaaattgggggagacgtg
```

```
ARTV/rat/GD    tgcatgcggggtccaaatcaactgagagcctcgtcgcggaatgccaaaggctgtgccagg
ARTVshrew/FS   tgctcttccgatctaaattgcccacatgcc----------------aaatttgtgtatgt

ARTV/rat/GD    tcgccctagatgctcctgccggatcaagccagattggggttaattcactcatggagaaat
ARTVshrew/FS   ttgttctggacaacattg-------------------------------------gaaat

ARTV/rat/GD    tgcttgaacccaccgaggaaagtgggtcaaagtcagagacacaagcca----------ta
ARTVshrew/FS   tgattgaaaagcaaaaagacaatgcgccgttgacctggacttgcgctagcggcatgtgtt

ARTV/rat/GD    atccaagcagccattgaggcgcttaaagcatgtaccc-----------------------
ARTVshrew/FS   tttctagcaagtgccaatgcccccaaagccctgccccattagatgcctttaatcatcagc

ARTV/rat/GD    -cgctgggcggttctacttgctcactcaccactgggcc-taatgacacttcctgggagac
ARTVshrew/FS   attttgagaaactatatttggccatcaaagatggtgccactatctcagctgctgtggccc

ARTV/rat/GD    aatccctcccagcgctgactcctctgatgtggacataccgaccctcgtggttg-------
ARTVshrew/FS   aaacttttccgtgccccaccccgctccctcgcacgtcctgtgctcgtcggtcggttacca

ARTV/rat/GD    aggctagctccaaggaaacacctaacgatgacataatcgttacggaacagcagccagctg
ARTVshrew/FS   acggaagctgctcagggatcctgggtggtgccatgaaggttgcgaaaccggattgtgcag

ARTV/rat/GD    aagtgcccgtgaaaccagttgcccctccacgtccaaagcggcaggcgctcaaagccgcgg
ARTVshrew/FS   agttggtgaccgacggacttgccgggttggtggagacgcaggtggcagtgggattggaag

ARTV/rat/GD    ccgttgcagattcagcaactcccatcaccatagggtctttgctttgggggacttccctcg
ARTVshrew/FS   cgaatgcaag--------------------------------------------------

ARTV/rat/GD    tcagtcagcaagctctcgaagttgtggagaagtcaaaggactgtttcacgatgttgactg
ARTVshrew/FS   -caacctgtcagaccctgtgcgcgagctgaaggtgacgacagattgtgcctcggagggtg

ARTV/rat/GD    gtaatgtgatgcgacgagggcagatccggaaaatgattgacacaattttgctccccgtga
ARTVshrew/FS   cgaacccaac--------------------------ttgtgccaattttgctccccgt--
```

```
ARTV/rat/GD     ccggtaaagacccagctgaagcttaccgcaacttgattgtggc--ggccaacacactgcc
ARTVshrew/FS    --------------------------------tcgactgttcctgtgccaaaccccgtg

ARTV/rat/GD     gacagtcttctcaaccggaaaggggcgttacttgcacatgcaga--------------ca
ARTVshrew/FS    cgaatttgccccaaaagagagtggtgtcaagagtgcctgtgggaaggacagcccggcca

ARTV/rat/GD     ccagttcaggtaaggaaccatgtcatagcaaacaaaccagacttcatcaacgcagagtct
ARTVshrew/FS    tcactgcatttactggacca----------------------------------------

ARTV/rat/GD     tgcgctcaccccaaaaatgtgcgcccaacctcagccgcggtggccgatattacaggggaa
ARTVshrew/FS    ----------acaacattgtgcgacgaatcgataccgc----------------tcgata

ARTV/rat/GD     tctgctcgggccaatcgctcaagatccggctctttgttttcgggctcctcagagactgtt
ARTVshrew/FS    tgtccgggaaacagccagtggatggtgacctcctttgtgaggtgtttttggaagatgctg

ARTV/rat/GD     tttgatgtccgaacaactgggcaaactag--------------tgagcggcgccaagagt
ARTVshrew/FS    atcacccatcaaacattcttagtaatttggccccctccaaatttaaaggtagccaaaccc

ARTV/rat/GD     cagtcgaagagcaaccacctcgacccgatcaggactctttagctctgagtggggtcgctg
ARTVshrew/FS    cgattcagccatccccccctcttgtgaacgggacatattcattgccagatg---------

ARTV/rat/GD     cgcctggcccaacatccaaacaggctcaaagcaatgtgactgaaaccccaactgtgggg
ARTVshrew/FS    ---------------------------------------tttacttgcctgattatgttt

ARTV/rat/GD     ctgaaactgaccaacctcctgggttaag--ggtcaagctaagggaggtgactgactcagt
ARTVshrew/FS    cagaatcttgcgatagtaatggatcgagatggtcagagttgaggagctggttgcgcaaac

ARTV/rat/GD     gacaaaacacttcgatgcgcaggttttccgcttgcactcccatctccttgccggccttaa
ARTVshrew/FS    ga-----------------ctggttttcatcaggcaaccccgcaccctt----gcttcaa

ARTV/rat/GD     cctcgtcgttaagccaactggaaagccatctggattggagtgtctctatcttctttggtg
ARTVshrew/FS    ttccacaggtggtatccatgctggctaaaccagggacagttcctctcaatttatttgact
```

```
ARTV/rat/GD     tctattgtgcattctccttggttttcatcacccggcctttgcttttctcccactgttggg
ARTVshrew/FS    ttttgggcatgttcattttgtgttttg----ccgggcttagtagcctggtggctcattgg

ARTV/rat/GD     aatggttactgggagtccttgggttcgtcgcgcttctctctttggcgtttggcttggggt
ARTVshrew/FS    ctttggctcctgtccttcttttgcttgttacttcccactcctagagggttactttacact

ARTV/rat/GD     tgcagttgttgtgttttcgccgacaaccagcgaaatgggggcatcttgccatgccgcttc
ARTVshrew/FS    tttattggctttctt-------------------------gggttccttgcttcttgcat

ARTV/rat/GD     accagagtgctcgacggctcttgcctattacacggattctgagcgtcataagcctgttca
ARTVshrew/FS    gcagcaccacagaaccctggacgtttgtgaccagggtttggagtcatgcaggcccatttt

ARTV/rat/GD     gc---------------------------------------ctgtgtctgttggcttttt
ARTVshrew/FS    gcaggacatggttgtgcatcgtgctactgctttggatccttttcccaccgtgggggcctt

ARTV/rat/GD     ggggaccctcggtgcagtacttgctgttgttctgggcgggcctcggtttttgtgggttct
ARTVshrew/FS    gggtgtattcctatccatcattgccaatttggttggtgggtccacggttttctattcttt

ARTV/rat/GD     tgtcctacgctgcctggtggtggtggacctgggatgcattgttgcctatgttttctttcg
ARTVshrew/FS    tgcctttaagcttgttcttatactggactgcgtactcattggcgtttggtattatttttc

ARTV/rat/GD     gcaaagatgcagacactgcttcaaaaattgtgtgagagttggcccgccggaaatcaattt
ARTVshrew/FS    aggagtttgtaccaaatgctggcgctattgcattagaacagcccatcgtgatgtgtccat

ARTV/rat/GD     gaagaccgtcccagtcactcgcgtggctagggggaccttagttagtttgtgtgatcagta
ARTVshrew/FS    gctgacgatcccagtcaccaaggtggccagaacagctcttaccacactatgcgaccaata

ARTV/rat/GD     cgcgcagccccatgttgatgtcgtccagctggccacaggaattccaggttgttggcgtgg
ARTVshrew/FS    ctgtcagcccccgttgacattgtggagatggccactggttattgtggttgctataccgg

ARTV/rat/GD     aaatgtcagtccacaccaggagtgttcaaaaccgatcccttacacagcttttgatgaaag
ARTVshrew/FS    --atctcagtctatgac-ggcgtatggcaaagccgtgccctatgctaagattgataaaaa
```

ARTV/rat/GD gaaggtcacccgccagactgttgtgccacaaccccacgatccaactcaagcagtgaaggc
ARTVshrew/FS caagctgggcctcgggaccatcattccattgccacaaaatgctactcaggcgagggacgc

ARTV/rat/GD cttaaaggtcttgatggcaaacggcagcttgcaggacgttgaaccaccgacagtcaaacc
ARTVshrew/FS aattagttttgtgtctcagggggcgtcattgctggttggcagccttccgcctgtgtccag

ARTV/rat/GD ggtcacgaaagtgccgtatgacgccccttctttccaaaggtgcccgtcgacccagagtg
ARTVshrew/FS agtttctaccattccatttaagtccccgtactttcccaatgttcctgtggatgagaccaa

ARTV/rat/GD cttcattgtggttgatgcagaaacttacaccgcagccttgcgttgtggatacagcaccaa
ARTVshrew/FS gcccattgtggttgaccaggctgtcta---ttcacgcttgtctgctgaacagagattgaa

ARTV/rat/GD gcctctgattgtcggcactggcaattttgccgaagtcaacaaaa----------------
ARTVshrew/FS g---gtggttcttggcatgggtgactttgtgcatgtgaatagaattttgccccctgaatt

ARTV/rat/GD --
ARTVshrew/FS gtcccccatttccaccacaatttggcctgaaaaagtgcttaccccggcaaggcgaagctg

ARTV/rat/GD ---cagtcagactgaccgggggggtttaaaggatttgatttgctcgcggtggctgtgtacg
ARTVshrew/FS gcttagcagcgcccaaattaaagttttaactgatgctggttatgccggtggctttatttg

ARTV/rat/GD tcatctgtaatttcttaattgtgtcctatttgtcatcacctctgt---------------
ARTVshrew/FS ctatcaattttatatttactggtctgcttgattttgggcctgttgctgcagcagcgtgg

ARTV/rat/GD --cttgcggagtgggcacgacggacccctggtgtcagcacccattcagcgttcccgtcta
ARTVshrew/FS tgtttgcggcattgacaccaacgataaattttgccgaaaccccttttcaaagccagttga

ARTV/rat/GD cggatcagggaccatgtgcaattctctcctttgtgtatcagctgaaggtctttcca----
ARTVshrew/FS ggggtatgctggatactgctatgccggttattgcctgtcatcaaaaggtcttagcgagtc

ARTV/rat/GD --------caccaattgccacttacgtcagcccgcaacgatggcttctggccatcgctct
ARTVshrew/FS tacacgaccactcgccgggatccctgtgagcatgattcagatctttttgctcgccgtttt

```
ARTV/rat/GD    gctttctacaactgtggttgtttgtctcagatttatatgctggcaggatttgttggcaat
ARTVshrew/FS   ggggctggctgggcttaccaaattgctgaaggttaccatttgggaacttttgctattgtt

ARTV/rat/GD    tgtcctcacgcttctaggttatgtgttcccgatcttgaacccaatttacctggcttttcc
ARTVshrew/FS   tttcccc------------------tttttaattttggtgtccccttggttggggttttt

ARTV/rat/GD    ttgcttggtagtttttgtgcaaatgcatccgctggtctttatttg------------gtc
ARTVshrew/FS   gaatgtgctagccattggctttttgcattttggggttaacacctatgtgggagcgatgca

ARTV/rat/GD    tcatttctttgtggcctctgttaatcccatggccgcagcatgcagcatcgtcatctgctt
ARTVshrew/FS   cctattgtttatttttctttttggaccctatgattggatttgttcaaattgccatgttgct

ARTV/rat/GD    tttggcttggatccttgggcggtcgaccggcgcagctggcttgattactccgtatgacat
ARTVshrew/FS   tgtgttggccatatttagatgggtcccgggagccccttttttggttacccccctttgattt

ARTV/rat/GD    ccactggttgccaccttcagcccaaaggagcgtggttaatgcgccgg------aaggttc
ARTVshrew/FS   ggctaaatttgtaaccaaccccaaaaatgctgctgccattgcccaggcctgtcctggtac

ARTV/rat/GD    gtacctcgctgccgtccgaagggccgccatcactgggcgcaacctgtttttctgccagtc
ARTVshrew/FS   ccatgcctttgccatgaaaacggctgcccttactggccgaaatttttcttttttgtgcccaa

ARTV/rat/GD    tagctttggcagcatcctcgagggtgcactaagaacatcagccagtcccacgaatgttgt
ARTVshrew/FS   tggttctggaatgatgtttgaaggccttcgccgcaaaccgattgtttcaaggaatgttgc

ARTV/rat/GD    gaccgtggtaggttcgtgcaccggctccgggggagtctttacactgcaaggaaagaaagt
ARTVshrew/FS   cactgtcgtcggttcgtcaactggagtgggttcgttgtggacacacaatggacaacagcg

ARTV/rat/GD    cgttatcacggcgtcacacctgttatgcgggttgcaggcccgcgtttccaaccacgagca
ARTVshrew/FS   tgtgtatacggctacccatctac---------------------ttgacgatgacaacaa

ARTV/rat/GD    gtcgatcctcgccaccttcacagtaaaaaatgactatgccttttctg-------------
ARTVshrew/FS   ggctgttgtgacattttgtggcgtaactaaagagctgacctttgatgtgcgtggcgattt
```

```
ARTV/rat/GD     --------aaatacctgaatggaaaggtccagcgcccccctgcaaaaatatctcgcaagag
ARTVshrew/FS    tgcccagtcaaattctaattttaatttcttagcccctgatcatgttccatccaaacctga

ARTV/rat/GD     gggccggtgtttctggttgactagcacgggcgttgagcctggggtggtgtccgaatcttc
ARTVshrew/FS    taacaaggcttattggttgaccaactccggtgtggaaggagggttctgtgatggcaccat

ARTV/rat/GD     agcactttgctttactcagtgcggtgactctggctcggtcgtggttgacgatgagaacaa
ARTVshrew/FS    ttgcgtttgcttcactgattgcggtgatagtggatcagctgtggtgaatagtgacgggca

ARTV/rat/GD     cgtggtaggagtgcacactggctccaacaagaaaggttgcggcatcatcacaatgcccga
ARTVshrew/FS    gtttatcggcgtgcacactggttccaacaaacagggtttggccgttatcaccaccccaga

ARTV/rat/GD     cggcacccccattggactcaccaacgtgaagttgtctgaattgtcaaaattcttcagcgg
ARTVshrew/FS    cggcaataccattggctatggtaaggtcaaattgtcaaaactttctcaattttattccgg

ARTV/rat/GD     gccactaatccctgtttcaaaactg---ccggaacacgtcatcaaggacaccgagctaat
ARTVshrew/FS    cccaatgacagctgtaccatcaccaattcccgataacattgaggcggattgtggtgaaat

ARTV/rat/GD     tccagcagacctagaggtggcgctcagacacaatgccacgcccgaaggagctctgtcatc
ARTVshrew/FS    cccttcttcattggctgatgccatcaacattaagatgaacaaggagggtgctctgccgac

ARTV/rat/GD     agtgcagcttttgtgtgtgtttgtgcttttgtggcgcttcatacatgtcccgatggttcc
ARTVshrew/FS    catttccatcttggtgactttcttggccttgtggagactgatgtttgttccctacggtgt

ARTV/rat/GD     tttcattgcagtgttctttttccttctaaatgagatactgccagtcacccttgtgcgtct
ARTVshrew/FS    accagttatgtgtggtttcttcttggccaatgaattgctgcctcagtggatgatgcgctg

ARTV/rat/GD     catcttctcgttcgcacttgggttggggtccctcttcactccgtactccgcaaatgtgct
ARTVshrew/FS    catttttgggatttttcttgattttgtgcaattggcctacgtacacccgggcgtcgtcat

ARTV/rat/GD     cctcattagattcctcacggcagcgctgaatcgcagtcttaagtcattgatgcttttcat
ARTVshrew/FS    tggcataaaattgctgacggcatctttgcacaagaatcaaaagtctttgctattttgggc
```

ARTV/rat/GD tgttctggctgttctttctggattgtccgagataatgctcaggcaaagcttgacctatgc
ARTVshrew/FS tgctgttgtttgcgctactttgatgattga--------------tggcccgaatactaac

ARTV/rat/GD cacctggttccttgttccgcggcagctggctattgctcattttcctgcagtcgtgggagt
ARTVshrew/FS ttttattcccttactttgttacttttgaatattattttcccgctt---------------

ARTV/rat/GD gttagcgggcgtgcatgtcacggcgctgttgcttagcatcttcaaattacactggctcac
ARTVshrew/FS -----------tactttcattgcacctgtttttggccctgtgcaggtggctgtgtcccgt

ARTV/rat/GD tgattgcttggca------ggcaatggcacttttgaaaaggccttctttatcaagtattt
ARTVshrew/FS ggtttgtgccacatactacggagaaggtgagtttagcagagcctttctgtgccgcatgct

ARTV/rat/GD tactgaaggaaacgtcagggatggggtgtcccagtcaaccaaacctgttgagggcctgac
ARTVshrew/FS ggcagagggcgctgaagggattcgaactggagtgacaaagagtggcatggcaagcccgga

ARTV/rat/GD cgctgggattgttgcctccctttcaa---------gtgaagacattgagtttgtccgacg
ARTVshrew/FS gtctttatctgcggcccttgccttaacgctcacgcatgacgagctgttgtttttggcgga

ARTV/rat/GD attcacagatgccaagtgttttgtgtcagcttcaaacatgcttaatggcgttaatgagtt
ARTVshrew/FS aaacaacgacgcgaagtgcgttaaatcagcatgcaaccttcaaaccgccgctcaaactta

ARTV/rat/GD tattgagtgtt------------gttacgccaaagccttgcgtgctgaagtctccaaagt
ARTVshrew/FS cattgaaacatcattttcaaaggctttgatcaaagcgttaagtggggtgtcctccaaggt

ARTV/rat/GD tgtgcagtgcaaaaataccacatcctgcctagcaaaattggaatcattcatttctggcac
ARTVshrew/FS ttcttgcaataa------------tttgcttgcaaaattagaagattttgttaccaacac

ARTV/rat/GD tcagtttgacctatcgcctggtgattgtgtggtcgtgatcggacgtgttccacgcgggga
ARTVshrew/FS ctccaccacacccaatccgggcgataaagtgattttcattggcaagttaccaaaatgtca

ARTV/rat/GD catcattgagatcacacacaaaggaagacccgtcgccttgcaggtgactgaaacgcgtgt
ARTVshrew/FS gattgttgagaaacatttgatggccaaacccgctcaatctttcttcaggaagaacgact

ARTV/rat/GD   catggccgggtcaccgtactcgatctgcac---ggttgtcacacctgagtctaccctgga
ARTVshrew/FS   ggtcgccggtacaatggtgaccattggcacctgcattgaaatgcaggggtctgccatcga

ARTV/rat/GD   gagctactatgggaaaggcaaatccaagaaaagattgcgcgcagaagcccgtggcatcaa
ARTVshrew/FS   ggtcaggtccatgaacggaaagaaagtgttgacggttaatggtaaacccgtaaacttcga

ARTV/rat/GD   ggtcaacagcagagttgtcgataaagtcaacattaacggcgttgagtacgttaaagtgtg
ARTVshrew/FS   ggatgatggcagagatcttggctctggtcgagttgacgagg--aataccttggaagcgtt

ARTV/rat/GD   gaataaagacacgggcgatattcattattactcacagcaggattatgatgattgggggaa
ARTVshrew/FS   gaggtcggtggcaagcagtattcaaatt--------------ctgggaccgtaaaactgg

ARTV/rat/GD   ggatgttcagtgggaaggcaccctgatttctgagaacccgcgtttcaacaccattggcat
ARTVshrew/FS   ggacgtctattatcgggacgcccaagatattacgatcggtaaatccaccatgactgctgg

ARTV/rat/GD   gtcgtcagctggccactttcaaaagttcatcaggaagcatgggaagaaagtagggacctg
ARTVshrew/FS   tctgcgcagagccctgaatgatgacatatttgccgagggtgatgagcccgtcaccgccgg

ARTV/rat/GD   cgtcaagaacatggccatctccaagaagaagtcagttgaagttgaactagagacgtacag
ARTVshrew/FS   agtcaagaggatgaccgtc---

ARTV/rat/GD   cctagatggagaagagtatgatattccaaagaacgagcctttggagtggactgtgacaat
ARTVshrew/FS   ----------------aacgaggccctgaatgcaatgggtttggattcgactatttctca

ARTV/rat/GD   ctacactgatgacaatgctgatgtttcgttggaggccgacagactgtcagttgagcaggc
ARTVshrew/FS   atctgatgttgctaaattggatgctttaattaa-------------taaattggttttgc

ARTV/rat/GD   tttgaatcaccttgggcatgacaccaaactcacagaaaaggaaaaagcccgcctcaaggc
ARTVshrew/FS   ttcgtgacacctc---

ARTV/rat/GD   catcatcgataaactgaatggattgtgccaggagaatgctttaaactgttag
ARTVshrew/FS   ---------------------------------aaagtgtttaaactcctaa

Table S2

The 5DS Differences (the pairwise differences in the combined sequence of the five domains of 3CLpro, NiRAN, RdRp, ZBD, and HEL1) between the viruses representing classified and unclassified genera and species of arteriviruses

The first virus	The second virus	5DS
Alphaarterivirus equid_X53459_EAV_horse	Betaarterivirus americense_AY150564_PRRSV-2_pig	61.36%
Alphaarterivirus equid_X53459_EAV_horse	Betaarterivirus americense_EF112445_PRRSV-2_pig	61.87%
Alphaarterivirus equid_X53459_EAV_horse	Betaarterivirus americense_JN654459_PRRSV-2__pig	61.62%
Alphaarterivirus equid_X53459_EAV_horse	Betaarterivirus americense_JQ308798_PRRSV-2_pig	61.36%
Alphaarterivirus equid_X53459_EAV_horse	Betaarterivirus americense_U87392_PRRSV-2_pig	61.36%
Alphaarterivirus equid_X53459_EAV_horse	Betaarterivirus americense_U87392_PRRSV-2_pig	60.61%
Alphaarterivirus equid_X53459_EAV_horse	Betaarterivirus europensis__KX668221_PRRSV-1_pig	61.87%
Alphaarterivirus equid_X53459_EAV_horse	Betaarterivirus europensis_JF802085_PRRSV-1_pig	62.28%
Alphaarterivirus equid_X53459_EAV_horse	Betaarterivirus europensis_M96262_PRRSV-1_pig	62.63%
Alphaarterivirus equid_X53459_EAV_horse	Betaarterivirus europensis_MT008024_PRRSV-1_pig	63.13%
Alphaarterivirus equid_X53459_EAV_horse	Betaarterivirus europensis_MT311646_PRRSV-1_pig	62.37%
Alphaarterivirus equid_X53459_EAV_horse	Betaarterivirus ninrav_KU302440_RtClonAV_rat	60.86%
Alphaarterivirus equid_X53459_EAV_horse	Betaarterivirus sheoin_KY369968_RtEiAV_rat	61.36%
Alphaarterivirus equid_X53459_EAV_horse	Betaarterivirus timiclar_KY369967_RtMcAV_rat	62.12%
Alphaarterivirus equid_X53459_EAV_horse	Deltaarterivirus hemfev_AF180391_SHFV_primate	64.30%
Alphaarterivirus equid_X53459_EAV_horse	Epsilonarterivirus hemcep_KM677927_SHEV_primate	63.22%
Alphaarterivirus equid_X53459_EAV_horse	Epsilonarterivirus safriver_KR862307_FSVV_primate	64.23%

Alphaarterivirus equid_X53459_EAV_horse	Epsilonarterivirus zamalb_KT166441_ZMbV-1_primate	65.49%
Alphaarterivirus equid_X53459_EAV_horse	Etaarterivirus ugarco_KC787658_KRCV-2_primate	64.81%
Alphaarterivirus equid_X53459_EAV_horse	Gammaarterivirus lacdeh_U15146_LDV_rat	62.03%
Alphaarterivirus equid_X53459_EAV_horse	Iotaarterivirus debrazmo_KP126831_DeBMAV_primate	64.05%
Alphaarterivirus equid_X53459_EAV_horse	Iotaarterivirus kibreg1_JX473849_KRTGV-1_primate	64.30%
Alphaarterivirus equid_X53459_EAV_horse	Iotaarterivirus pejah_KR139839_PBJV_primate	66.33%
Alphaarterivirus equid_X53459_EAV_horse	Kappaarterivirus wobum_JN116253_WPDV_possum	66.33%
Alphaarterivirus equid_X53459_EAV_horse	Lambdaarterivirus afriporav_KP026921_APRAV_rat	65.08%
Alphaarterivirus equid_X53459_EAV_horse	Muarterivirus afrigant_MF324848_OSV-1_shrew	63.66%
Alphaarterivirus equid_X53459_EAV_horse	Muarterivirus afrigant_MF324849_OSV-2_shrew	63.66%
Alphaarterivirus equid_X53459_EAV_horse	Muarterivirus afrigant_MG264317_OSV-3_shrew	63.66%
Alphaarterivirus equid_X53459_EAV_horse	Nuarterivirus guemel_KY369969_RtClanAV_rat	60.00%
Alphaarterivirus equid_X53459_EAV_horse	PP947442_ARTV/shrew/FS_shrew	62.92%
Alphaarterivirus equid_X53459_EAV_horse	PP964217_ARTV/rat/GD_rat	64.05%
Alphaarterivirus equid_X53459_EAV_horse	Thetaarterivirus kafuba_KT447550_KKCBV_primate	63.71%
Alphaarterivirus equid_X53459_EAV_horse	Thetaarterivirus mikelba1_KM110938_MYBV-1_primate	60.91%
Alphaarterivirus equid_X53459_EAV_horse	Unclassified_KP280007_RtDs-Arterivirus -4_rat	59.75%
Alphaarterivirus equid_X53459_EAV_horse	Unclassified_MT085132_RtRe-ArtV-Tka2010B_rat	64.30%
Alphaarterivirus equid_X53459_EAV_horse	Unclassified_MT085133_RtRe-ArtV-Tka2010C_rat	63.80%
Alphaarterivirus equid_X53459_EAV_horse	Unclassified_MT085136_RtRe-ArtV-Tl2006A_rat	63.54%
Alphaarterivirus equid_X53459_EAV_horse	Unclassified_OQ715736_JSV-1_shrew	62.37%
Alphaarterivirus equid_X53459_EAV_horse	Zetaarterivirus ugarco_KC787630_KRCV-1_primate	64.39%
Betaarterivirus americense_AY150564_PRRSV-2_pig	Betaarterivirus americense_EF112445_PRRSV-2_pig	3.25%
Betaarterivirus americense_AY150564_PRRSV-2_pig	Betaarterivirus americense_JN654459_PRRSV-2__pig	4.50%

Betaarterivirus americense_AY150564_PRRSV-2_pig	Betaarterivirus americense_JQ308798_PRRSV-2_pig	5.25%
Betaarterivirus americense_AY150564_PRRSV-2_pig	Betaarterivirus americense_U87392_PRRSV-2_pig	0.00%
Betaarterivirus americense_AY150564_PRRSV-2_pig	Betaarterivirus chinrav_KP280006_RtMrufAV_rat	24.50%
Betaarterivirus americense_AY150564_PRRSV-2_pig	Betaarterivirus europensis__KX668221_PRRSV-1_pig	34.09%
Betaarterivirus americense_AY150564_PRRSV-2_pig	Betaarterivirus europensis_JF802085_PRRSV-1_pig	35.18%
Betaarterivirus americense_AY150564_PRRSV-2_pig	Betaarterivirus europensis_M96262_PRRSV-1_pig	33.83%
Betaarterivirus americense_AY150564_PRRSV-2_pig	Betaarterivirus europensis_MT008024_PRRSV-1_pig	33.58%
Betaarterivirus americense_AY150564_PRRSV-2_pig	Betaarterivirus europensis_MT311646_PRRSV-1_pig	33.58%
Betaarterivirus americense_AY150564_PRRSV-2_pig	Betaarterivirus ninrav_KU302440_RtClonAV_rat	26.50%
Betaarterivirus americense_AY150564_PRRSV-2_pig	Betaarterivirus sheoin_KY369968_RtEiAV_rat	26.50%
Betaarterivirus americense_AY150564_PRRSV-2_pig	Betaarterivirus timiclar_KY369967_RtMcAV_rat	26.50%
Betaarterivirus americense_AY150564_PRRSV-2_pig	Deltaarterivirus hemfev_AF180391_SHFV_primate	53.27%
Betaarterivirus americense_AY150564_PRRSV-2_pig	Epsilonarterivirus hemcep_KM677927_SHEV_primate	51.26%
Betaarterivirus americense_AY150564_PRRSV-2_pig	Epsilonarterivirus safriver_KR862307_FSVV_primate	50.25%
Betaarterivirus americense_AY150564_PRRSV-2_pig	Epsilonarterivirus zamalb_KT166441_ZMbV-1_primate	49.50%
Betaarterivirus americense_AY150564_PRRSV-2_pig	Etaarterivirus ugarco_KC787658_KRCV-2_primate	53.90%
Betaarterivirus americense_AY150564_PRRSV-2_pig	Gammaarterivirus lacdeh_U15146_LDV_rat	45.73%
Betaarterivirus americense_AY150564_PRRSV-2_pig	Iotaarterivirus debrazmo_KP126831_DeBMAV_primate	56.78%
Betaarterivirus americense_AY150564_PRRSV-2_pig	Iotaarterivirus kibreg1_JX473849_KRTGV-1_primate	56.03%
Betaarterivirus americense_AY150564_PRRSV-2_pig	Iotaarterivirus pejah_KR139839_PBJV_primate	56.03%
Betaarterivirus americense_AY150564_PRRSV-2_pig	Kappaarterivirus wobum_JN116253_WPDV_possum	69.29%
Betaarterivirus americense_AY150564_PRRSV-2_pig	Lambdaarterivirus afriporav_KP026921_APRAV_rat	58.04%
Betaarterivirus americense_AY150564_PRRSV-2_pig	Muarterivirus afrigant_MF324848_OSV-1_shrew	63.66%
Betaarterivirus americense_AY150564_PRRSV-2_pig	Muarterivirus afrigant_MF324849_OSV-2_shrew	63.40%

Betaarterivirus americense_AY150564_PRRSV-2_pig	Muarterivirus afrigant_MG264317_OSV-3_shrew	62.63%
Betaarterivirus americense_AY150564_PRRSV-2_pig	Nuarterivirus guemel_KY369969_RtClanAV_rat	37.69%
Betaarterivirus americense_AY150564_PRRSV-2_pig	Thetaarterivirus kafuba_KT447550_KKCBV_primate	54.91%
Betaarterivirus americense_AY150564_PRRSV-2_pig	Thetaarterivirus mikelba1_KM110938_MYBV-1_primate	54.91%
Betaarterivirus americense_AY150564_PRRSV-2_pig	Unclassified_KP280007_RtDs-Arterivirus -4_rat	42.96%
Betaarterivirus americense_AY150564_PRRSV-2_pig	Unclassified_MT085132_RtRe-ArtV-Tka2010B_rat	50.75%
Betaarterivirus americense_AY150564_PRRSV-2_pig	Unclassified_MT085133_RtRe-ArtV-Tka2010C_rat	50.50%
Betaarterivirus americense_AY150564_PRRSV-2_pig	Unclassified_MT085136_RtRe-ArtV-Tl2006A_rat	51.26%
Betaarterivirus americense_AY150564_PRRSV-2_pig	Unclassified_OQ715736_JSV-1_shrew	61.34%
Betaarterivirus americense_AY150564_PRRSV-2_pig	Zetaarterivirus ugarco_KC787630_KRCV-1_primate	54.52%
Betaarterivirus americense_EF112445_PRRSV-2_pig	Betaarterivirus americense_JN654459_PRRSV-2__pig	4.25%
Betaarterivirus americense_EF112445_PRRSV-2_pig	Betaarterivirus americense_JQ308798_PRRSV-2_pig	3.50%
Betaarterivirus americense_EF112445_PRRSV-2_pig	Betaarterivirus europensis__KX668221_PRRSV-1_pig	33.33%
Betaarterivirus americense_EF112445_PRRSV-2_pig	Betaarterivirus europensis_JF802085_PRRSV-1_pig	34.92%
Betaarterivirus americense_EF112445_PRRSV-2_pig	Betaarterivirus europensis_MT008024_PRRSV-1_pig	32.58%
Betaarterivirus americense_EF112445_PRRSV-2_pig	Betaarterivirus europensis_MT311646_PRRSV-1_pig	32.83%
Betaarterivirus americense_EF112445_PRRSV-2_pig	Epsilonarterivirus hemcep_KM677927_SHEV_primate	51.51%
Betaarterivirus americense_EF112445_PRRSV-2_pig	Epsilonarterivirus safriver_KR862307_FSVV_primate	50.25%
Betaarterivirus americense_EF112445_PRRSV-2_pig	Epsilonarterivirus zamalb_KT166441_ZMbV-1_primate	50.00%
Betaarterivirus americense_EF112445_PRRSV-2_pig	Etaarterivirus ugarco_KC787658_KRCV-2_primate	54.41%
Betaarterivirus americense_EF112445_PRRSV-2_pig	Gammaarterivirus lacdeh_U15146_LDV_rat	45.98%
Betaarterivirus americense_EF112445_PRRSV-2_pig	Iotaarterivirus debrazmo_KP126831_DeBMAV_primate	56.28%
Betaarterivirus americense_EF112445_PRRSV-2_pig	Iotaarterivirus kibreg1_JX473849_KRTGV-1_primate	56.28%
Betaarterivirus americense_EF112445_PRRSV-2_pig	Iotaarterivirus pejah_KR139839_PBJV_primate	56.28%

Betaarterivirus americense_EF112445_PRRSV-2_pig	Kappaarterivirus wobum_JN116253_WPDV_possum	69.80%
Betaarterivirus americense_EF112445_PRRSV-2_pig	Lambdaarterivirus afriporav_KP026921_APRAV_rat	58.54%
Betaarterivirus americense_EF112445_PRRSV-2_pig	Muarterivirus afrigant_MF324848_OSV-1_shrew	62.63%
Betaarterivirus americense_EF112445_PRRSV-2_pig	Muarterivirus afrigant_MF324849_OSV-2_shrew	62.37%
Betaarterivirus americense_EF112445_PRRSV-2_pig	Muarterivirus afrigant_MG264317_OSV-3_shrew	62.11%
Betaarterivirus americense_EF112445_PRRSV-2_pig	Nuarterivirus guemel_KY369969_RtClanAV_rat	37.94%
Betaarterivirus americense_EF112445_PRRSV-2_pig	Thetaarterivirus kafuba_KT447550_KKCBV_primate	54.91%
Betaarterivirus americense_EF112445_PRRSV-2_pig	Thetaarterivirus mikelba1_KM110938_MYBV-1_primate	54.91%
Betaarterivirus americense_EF112445_PRRSV-2_pig	Unclassified_KP280007_RtDs-Arterivirus -4_rat	42.96%
Betaarterivirus americense_EF112445_PRRSV-2_pig	Unclassified_MT085132_RtRe-ArtV-Tka2010B_rat	50.00%
Betaarterivirus americense_EF112445_PRRSV-2_pig	Unclassified_MT085133_RtRe-ArtV-Tka2010C_rat	49.75%
Betaarterivirus americense_EF112445_PRRSV-2_pig	Unclassified_MT085136_RtRe-ArtV-Tl2006A_rat	50.25%
Betaarterivirus americense_EF112445_PRRSV-2_pig	Unclassified_OQ715736_JSV-1_shrew	61.08%
Betaarterivirus americense_EF112445_PRRSV-2_pig	Zetaarterivirus ugarco_KC787630_KRCV-1_primate	54.77%
Betaarterivirus americense_JN654459_PRRSV-2__pig	Betaarterivirus americense_JQ308798_PRRSV-2_pig	5.75%
Betaarterivirus americense_JN654459_PRRSV-2__pig	Betaarterivirus europensis__KX668221_PRRSV-1_pig	34.09%
Betaarterivirus americense_JN654459_PRRSV-2__pig	Betaarterivirus europensis_MT008024_PRRSV-1_pig	32.58%
Betaarterivirus americense_JN654459_PRRSV-2__pig	Betaarterivirus europensis_MT311646_PRRSV-1_pig	33.08%
Betaarterivirus americense_JN654459_PRRSV-2__pig	Kappaarterivirus wobum_JN116253_WPDV_possum	69.54%
Betaarterivirus americense_JN654459_PRRSV-2__pig	Lambdaarterivirus afriporav_KP026921_APRAV_rat	58.04%
Betaarterivirus americense_JN654459_PRRSV-2__pig	Muarterivirus afrigant_MF324848_OSV-1_shrew	62.37%
Betaarterivirus americense_JN654459_PRRSV-2__pig	Muarterivirus afrigant_MF324849_OSV-2_shrew	62.11%
Betaarterivirus americense_JN654459_PRRSV-2__pig	Muarterivirus afrigant_MG264317_OSV-3_shrew	61.86%
Betaarterivirus americense_JN654459_PRRSV-2__pig	Nuarterivirus guemel_KY369969_RtClanAV_rat	37.69%

Betaarterivirus americense_JN654459_PRRSV-2__pig	Thetaarterivirus kafuba_KT447550_KKCBV_primate	54.41%
Betaarterivirus americense_JN654459_PRRSV-2__pig	Thetaarterivirus mikelba1_KM110938_MYBV-1_primate	53.90%
Betaarterivirus americense_JN654459_PRRSV-2__pig	Unclassified_KP280007_RtDs-Arterivirus -4_rat	42.96%
Betaarterivirus americense_JN654459_PRRSV-2__pig	Unclassified_MT085132_RtRe-ArtV-Tka2010B_rat	51.01%
Betaarterivirus americense_JN654459_PRRSV-2__pig	Unclassified_MT085133_RtRe-ArtV-Tka2010C_rat	50.75%
Betaarterivirus americense_JN654459_PRRSV-2__pig	Unclassified_MT085136_RtRe-ArtV-Tl2006A_rat	50.75%
Betaarterivirus americense_JN654459_PRRSV-2__pig	Unclassified_OQ715736_JSV-1_shrew	60.82%
Betaarterivirus americense_JN654459_PRRSV-2__pig	Zetaarterivirus ugarco_KC787630_KRCV-1_primate	54.52%
Betaarterivirus americense_JQ308798_PRRSV-2_pig	Betaarterivirus europensis__KX668221_PRRSV-1_pig	33.08%
Betaarterivirus americense_JQ308798_PRRSV-2_pig	Betaarterivirus europensis_MT008024_PRRSV-1_pig	32.58%
Betaarterivirus americense_JQ308798_PRRSV-2_pig	Betaarterivirus europensis_MT311646_PRRSV-1_pig	32.33%
Betaarterivirus americense_JQ308798_PRRSV-2_pig	Kappaarterivirus wobum_JN116253_WPDV_possum	69.54%
Betaarterivirus americense_JQ308798_PRRSV-2_pig	Lambdaarterivirus afriporav_KP026921_APRAV_rat	58.79%
Betaarterivirus americense_JQ308798_PRRSV-2_pig	Muarterivirus afrigant_MF324848_OSV-1_shrew	63.14%
Betaarterivirus americense_JQ308798_PRRSV-2_pig	Muarterivirus afrigant_MF324849_OSV-2_shrew	62.89%
Betaarterivirus americense_JQ308798_PRRSV-2_pig	Muarterivirus afrigant_MG264317_OSV-3_shrew	62.63%
Betaarterivirus americense_JQ308798_PRRSV-2_pig	Nuarterivirus guemel_KY369969_RtClanAV_rat	38.44%
Betaarterivirus americense_JQ308798_PRRSV-2_pig	Thetaarterivirus kafuba_KT447550_KKCBV_primate	55.16%
Betaarterivirus americense_JQ308798_PRRSV-2_pig	Thetaarterivirus mikelba1_KM110938_MYBV-1_primate	55.42%
Betaarterivirus americense_JQ308798_PRRSV-2_pig	Unclassified_KP280007_RtDs-Arterivirus -4_rat	43.47%
Betaarterivirus americense_JQ308798_PRRSV-2_pig	Unclassified_MT085132_RtRe-ArtV-Tka2010B_rat	51.01%
Betaarterivirus americense_JQ308798_PRRSV-2_pig	Unclassified_MT085133_RtRe-ArtV-Tka2010C_rat	50.75%
Betaarterivirus americense_JQ308798_PRRSV-2_pig	Unclassified_MT085136_RtRe-ArtV-Tl2006A_rat	51.01%
Betaarterivirus americense_JQ308798_PRRSV-2_pig	Unclassified_OQ715736_JSV-1_shrew	61.08%

Betaarterivirus americense_JQ308798_PRRSV-2_pig	Zetaarterivirus ugarco_KC787630_KRCV-1_primate	54.77%
Betaarterivirus americense_U87392_PRRSV-2_pig	Betaarterivirus americense_EF112445_PRRSV-2_pig	3.25%
Betaarterivirus americense_U87392_PRRSV-2_pig	Betaarterivirus americense_JN654459_PRRSV-2__pig	4.50%
Betaarterivirus americense_U87392_PRRSV-2_pig	Betaarterivirus americense_JQ308798_PRRSV-2_pig	5.25%
Betaarterivirus americense_U87392_PRRSV-2_pig	Betaarterivirus chinrav_KP280006_RtMrufAV_rat	24.50%
Betaarterivirus americense_U87392_PRRSV-2_pig	Betaarterivirus europensis__KX668221_PRRSV-1_pig	34.09%
Betaarterivirus americense_U87392_PRRSV-2_pig	Betaarterivirus europensis_JF802085_PRRSV-1_pig	35.18%
Betaarterivirus americense_U87392_PRRSV-2_pig	Betaarterivirus europensis_M96262_PRRSV-1_pig	33.83%
Betaarterivirus americense_U87392_PRRSV-2_pig	Betaarterivirus europensis_MT008024_PRRSV-1_pig	33.58%
Betaarterivirus americense_U87392_PRRSV-2_pig	Betaarterivirus europensis_MT311646_PRRSV-1_pig	33.58%
Betaarterivirus americense_U87392_PRRSV-2_pig	Betaarterivirus ninrav_KU302440_RtClonAV_rat	26.50%
Betaarterivirus americense_U87392_PRRSV-2_pig	Betaarterivirus sheoin_KY369968_RtEiAV_rat	26.50%
Betaarterivirus americense_U87392_PRRSV-2_pig	Betaarterivirus timiclar_KY369967_RtMcAV_rat	26.50%
Betaarterivirus americense_U87392_PRRSV-2_pig	Deltaarterivirus hemfev_AF180391_SHFV_primate	53.27%
Betaarterivirus americense_U87392_PRRSV-2_pig	Epsilonarterivirus hemcep_KM677927_SHEV_primate	51.26%
Betaarterivirus americense_U87392_PRRSV-2_pig	Epsilonarterivirus safriver_KR862307_FSVV_primate	50.25%
Betaarterivirus americense_U87392_PRRSV-2_pig	Epsilonarterivirus zamalb_KT166441_ZMbV-1_primate	49.50%
Betaarterivirus americense_U87392_PRRSV-2_pig	Etaarterivirus ugarco_KC787658_KRCV-2_primate	53.90%
Betaarterivirus americense_U87392_PRRSV-2_pig	Gammaarterivirus lacdeh_U15146_LDV_rat	45.73%
Betaarterivirus americense_U87392_PRRSV-2_pig	Iotaarterivirus debrazmo_KP126831_DeBMAV_primate	56.78%
Betaarterivirus americense_U87392_PRRSV-2_pig	Iotaarterivirus kibreg1_JX473849_KRTGV-1_primate	56.03%
Betaarterivirus americense_U87392_PRRSV-2_pig	Iotaarterivirus pejah_KR139839_PBJV_primate	56.03%
Betaarterivirus americense_U87392_PRRSV-2_pig	Kappaarterivirus wobum_JN116253_WPDV_possum	69.29%
Betaarterivirus americense_U87392_PRRSV-2_pig	Lambdaarterivirus afriporav_KP026921_APRAV_rat	58.04%

Betaarterivirus americense_U87392_PRRSV-2_pig	Muarterivirus afrigant_MF324848_OSV-1_shrew	63.66%
Betaarterivirus americense_U87392_PRRSV-2_pig	Muarterivirus afrigant_MF324849_OSV-2_shrew	63.40%
Betaarterivirus americense_U87392_PRRSV-2_pig	Muarterivirus afrigant_MG264317_OSV-3_shrew	62.63%
Betaarterivirus americense_U87392_PRRSV-2_pig	Nuarterivirus guemel_KY369969_RtClanAV_rat	37.69%
Betaarterivirus americense_U87392_PRRSV-2_pig	Thetaarterivirus kafuba_KT447550_KKCBV_primate	54.91%
Betaarterivirus americense_U87392_PRRSV-2_pig	Thetaarterivirus mikelba1_KM110938_MYBV-1_primate	54.91%
Betaarterivirus americense_U87392_PRRSV-2_pig	Unclassified_KP280007_RtDs-Arterivirus -4_rat	42.96%
Betaarterivirus americense_U87392_PRRSV-2_pig	Unclassified_MT085132_RtRe-ArtV-Tka2010B_rat	50.75%
Betaarterivirus americense_U87392_PRRSV-2_pig	Unclassified_MT085133_RtRe-ArtV-Tka2010C_rat	50.50%
Betaarterivirus americense_U87392_PRRSV-2_pig	Unclassified_MT085136_RtRe-ArtV-Tl2006A_rat	51.26%
Betaarterivirus americense_U87392_PRRSV-2_pig	Unclassified_OQ715736_JSV-1_shrew	61.34%
Betaarterivirus americense_U87392_PRRSV-2_pig	Zetaarterivirus ugarco_KC787630_KRCV-1_primate	54.52%
Betaarterivirus chinrav_KP280006_RtMrufAV_rat	Betaarterivirus americense_EF112445_PRRSV-2_pig	23.25%
Betaarterivirus chinrav_KP280006_RtMrufAV_rat	Betaarterivirus americense_JN654459_PRRSV-2__pig	24.75%
Betaarterivirus chinrav_KP280006_RtMrufAV_rat	Betaarterivirus americense_JQ308798_PRRSV-2_pig	23.50%
Betaarterivirus chinrav_KP280006_RtMrufAV_rat	Betaarterivirus europensis__KX668221_PRRSV-1_pig	31.58%
Betaarterivirus chinrav_KP280006_RtMrufAV_rat	Betaarterivirus europensis_JF802085_PRRSV-1_pig	32.41%
Betaarterivirus chinrav_KP280006_RtMrufAV_rat	Betaarterivirus europensis_M96262_PRRSV-1_pig	31.58%
Betaarterivirus chinrav_KP280006_RtMrufAV_rat	Betaarterivirus europensis_MT008024_PRRSV-1_pig	31.58%
Betaarterivirus chinrav_KP280006_RtMrufAV_rat	Betaarterivirus europensis_MT311646_PRRSV-1_pig	31.33%
Betaarterivirus chinrav_KP280006_RtMrufAV_rat	Betaarterivirus ninrav_KU302440_RtClonAV_rat	17.00%
Betaarterivirus chinrav_KP280006_RtMrufAV_rat	Betaarterivirus sheoin_KY369968_RtEiAV_rat	20.25%
Betaarterivirus chinrav_KP280006_RtMrufAV_rat	Betaarterivirus timiclar_KY369967_RtMcAV_rat	25.50%
Betaarterivirus chinrav_KP280006_RtMrufAV_rat	Deltaarterivirus hemfev_AF180391_SHFV_primate	53.77%

Betaarterivirus chinrav_KP280006_RtMrufAV_rat	Epsilonarterivirus hemcep_KM677927_SHEV_primate	53.02%
Betaarterivirus chinrav_KP280006_RtMrufAV_rat	Epsilonarterivirus safriver_KR862307_FSVV_primate	51.51%
Betaarterivirus chinrav_KP280006_RtMrufAV_rat	Epsilonarterivirus zamalb_KT166441_ZMbV-1_primate	50.00%
Betaarterivirus chinrav_KP280006_RtMrufAV_rat	Etaarterivirus ugarco_KC787658_KRCV-2_primate	54.41%
Betaarterivirus chinrav_KP280006_RtMrufAV_rat	Gammaarterivirus lacdeh_U15146_LDV_rat	44.72%
Betaarterivirus chinrav_KP280006_RtMrufAV_rat	Iotaarterivirus debrazmo_KP126831_DeBMAV_primate	53.27%
Betaarterivirus chinrav_KP280006_RtMrufAV_rat	Iotaarterivirus kibreg1_JX473849_KRTGV-1_primate	56.03%
Betaarterivirus chinrav_KP280006_RtMrufAV_rat	Iotaarterivirus pejah_KR139839_PBJV_primate	56.28%
Betaarterivirus chinrav_KP280006_RtMrufAV_rat	Kappaarterivirus wobum_JN116253_WPDV_possum	69.29%
Betaarterivirus chinrav_KP280006_RtMrufAV_rat	Lambdaarterivirus afriporav_KP026921_APRAV_rat	59.80%
Betaarterivirus chinrav_KP280006_RtMrufAV_rat	Muarterivirus afrigant_MF324848_OSV-1_shrew	62.37%
Betaarterivirus chinrav_KP280006_RtMrufAV_rat	Muarterivirus afrigant_MF324849_OSV-2_shrew	61.86%
Betaarterivirus chinrav_KP280006_RtMrufAV_rat	Muarterivirus afrigant_MG264317_OSV-3_shrew	61.86%
Betaarterivirus chinrav_KP280006_RtMrufAV_rat	Nuarterivirus guemel_KY369969_RtClanAV_rat	36.93%
Betaarterivirus chinrav_KP280006_RtMrufAV_rat	Thetaarterivirus kafuba_KT447550_KKCBV_primate	55.42%
Betaarterivirus chinrav_KP280006_RtMrufAV_rat	Thetaarterivirus mikelba1_KM110938_MYBV-1_primate	55.67%
Betaarterivirus chinrav_KP280006_RtMrufAV_rat	Unclassified_KP280007_RtDs-Arterivirus -4_rat	41.46%
Betaarterivirus chinrav_KP280006_RtMrufAV_rat	Unclassified_MT085132_RtRe-ArtV-Tka2010B_rat	50.25%
Betaarterivirus chinrav_KP280006_RtMrufAV_rat	Unclassified_MT085133_RtRe-ArtV-Tka2010C_rat	50.75%
Betaarterivirus chinrav_KP280006_RtMrufAV_rat	Unclassified_MT085136_RtRe-ArtV-Tl2006A_rat	50.00%
Betaarterivirus chinrav_KP280006_RtMrufAV_rat	Unclassified_OQ715736_JSV-1_shrew	60.05%
Betaarterivirus chinrav_KP280006_RtMrufAV_rat	Zetaarterivirus ugarco_KC787630_KRCV-1_primate	53.27%
Betaarterivirus europensis__KX668221_PRRSV-1_pig	Betaarterivirus europensis_MT008024_PRRSV-1_pig	8.02%
Betaarterivirus europensis__KX668221_PRRSV-1_pig	Betaarterivirus europensis_MT311646_PRRSV-1_pig	9.02%

Betaarterivirus europensis__KX668221_PRRSV-1_pig	Lambdaarterivirus afriporav_KP026921_APRAV_rat	58.04%
Betaarterivirus europensis__KX668221_PRRSV-1_pig	Muarterivirus afrigant_MF324848_OSV-1_shrew	60.31%
Betaarterivirus europensis__KX668221_PRRSV-1_pig	Muarterivirus afrigant_MF324849_OSV-2_shrew	59.79%
Betaarterivirus europensis__KX668221_PRRSV-1_pig	Muarterivirus afrigant_MG264317_OSV-3_shrew	59.02%
Betaarterivirus europensis__KX668221_PRRSV-1_pig	Nuarterivirus guemel_KY369969_RtClanAV_rat	37.19%
Betaarterivirus europensis__KX668221_PRRSV-1_pig	Thetaarterivirus kafuba_KT447550_KKCBV_primate	50.51%
Betaarterivirus europensis__KX668221_PRRSV-1_pig	Thetaarterivirus mikelba1_KM110938_MYBV-1_primate	52.78%
Betaarterivirus europensis__KX668221_PRRSV-1_pig	Unclassified_MT085132_RtRe-ArtV-Tka2010B_rat	47.24%
Betaarterivirus europensis__KX668221_PRRSV-1_pig	Unclassified_MT085133_RtRe-ArtV-Tka2010C_rat	47.49%
Betaarterivirus europensis__KX668221_PRRSV-1_pig	Unclassified_MT085136_RtRe-ArtV-Tl2006A_rat	48.24%
Betaarterivirus europensis__KX668221_PRRSV-1_pig	Unclassified_OQ715736_JSV-1_shrew	58.51%
Betaarterivirus europensis__KX668221_PRRSV-1_pig	Zetaarterivirus ugarco_KC787630_KRCV-1_primate	53.15%
Betaarterivirus europensis_JF802085_PRRSV-1_pig	Betaarterivirus americense_JN654459_PRRSV-2__pig	35.43%
Betaarterivirus europensis_JF802085_PRRSV-1_pig	Betaarterivirus americense_JQ308798_PRRSV-2_pig	34.67%
Betaarterivirus europensis_JF802085_PRRSV-1_pig	Betaarterivirus europensis__KX668221_PRRSV-1_pig	8.79%
Betaarterivirus europensis_JF802085_PRRSV-1_pig	Betaarterivirus europensis_MT008024_PRRSV-1_pig	12.06%
Betaarterivirus europensis_JF802085_PRRSV-1_pig	Betaarterivirus europensis_MT311646_PRRSV-1_pig	10.30%
Betaarterivirus europensis_JF802085_PRRSV-1_pig	Kappaarterivirus wobum_JN116253_WPDV_possum	69.64%
Betaarterivirus europensis_JF802085_PRRSV-1_pig	Lambdaarterivirus afriporav_KP026921_APRAV_rat	57.93%
Betaarterivirus europensis_JF802085_PRRSV-1_pig	Muarterivirus afrigant_MF324848_OSV-1_shrew	61.24%
Betaarterivirus europensis_JF802085_PRRSV-1_pig	Muarterivirus afrigant_MF324849_OSV-2_shrew	60.98%
Betaarterivirus europensis_JF802085_PRRSV-1_pig	Muarterivirus afrigant_MG264317_OSV-3_shrew	60.47%
Betaarterivirus europensis_JF802085_PRRSV-1_pig	Nuarterivirus guemel_KY369969_RtClanAV_rat	38.04%
Betaarterivirus europensis_JF802085_PRRSV-1_pig	Thetaarterivirus kafuba_KT447550_KKCBV_primate	52.41%

Betaarterivirus europensis_JF802085_PRRSV-1_pig	Thetaarterivirus mikelba1_KM110938_MYBV-1_primate	54.68%
Betaarterivirus europensis_JF802085_PRRSV-1_pig	Unclassified_KP280007_RtDs-Arterivirus -4_rat	40.55%
Betaarterivirus europensis_JF802085_PRRSV-1_pig	Unclassified_MT085132_RtRe-ArtV-Tka2010B_rat	48.11%
Betaarterivirus europensis_JF802085_PRRSV-1_pig	Unclassified_MT085133_RtRe-ArtV-Tka2010C_rat	48.36%
Betaarterivirus europensis_JF802085_PRRSV-1_pig	Unclassified_MT085136_RtRe-ArtV-Tl2006A_rat	48.36%
Betaarterivirus europensis_JF802085_PRRSV-1_pig	Unclassified_OQ715736_JSV-1_shrew	60.72%
Betaarterivirus europensis_JF802085_PRRSV-1_pig	Zetaarterivirus ugarco_KC787630_KRCV-1_primate	53.03%
Betaarterivirus europensis_M96262_PRRSV-1_pig	Betaarterivirus americense_EF112445_PRRSV-2_pig	33.08%
Betaarterivirus europensis_M96262_PRRSV-1_pig	Betaarterivirus americense_JN654459_PRRSV-2__pig	33.33%
Betaarterivirus europensis_M96262_PRRSV-1_pig	Betaarterivirus americense_JQ308798_PRRSV-2_pig	32.58%
Betaarterivirus europensis_M96262_PRRSV-1_pig	Betaarterivirus europensis__KX668221_PRRSV-1_pig	8.77%
Betaarterivirus europensis_M96262_PRRSV-1_pig	Betaarterivirus europensis_JF802085_PRRSV-1_pig	10.05%
Betaarterivirus europensis_M96262_PRRSV-1_pig	Betaarterivirus europensis_MT008024_PRRSV-1_pig	7.52%
Betaarterivirus europensis_M96262_PRRSV-1_pig	Betaarterivirus europensis_MT311646_PRRSV-1_pig	0.25%
Betaarterivirus europensis_M96262_PRRSV-1_pig	Betaarterivirus ninrav_KU302440_RtClonAV_rat	31.33%
Betaarterivirus europensis_M96262_PRRSV-1_pig	Betaarterivirus sheoin_KY369968_RtEiAV_rat	31.08%
Betaarterivirus europensis_M96262_PRRSV-1_pig	Betaarterivirus timiclar_KY369967_RtMcAV_rat	31.83%
Betaarterivirus europensis_M96262_PRRSV-1_pig	Deltaarterivirus hemfev_AF180391_SHFV_primate	53.15%
Betaarterivirus europensis_M96262_PRRSV-1_pig	Epsilonarterivirus hemcep_KM677927_SHEV_primate	51.64%
Betaarterivirus europensis_M96262_PRRSV-1_pig	Epsilonarterivirus safriver_KR862307_FSVV_primate	50.38%
Betaarterivirus europensis_M96262_PRRSV-1_pig	Epsilonarterivirus zamalb_KT166441_ZMbV-1_primate	51.13%
Betaarterivirus europensis_M96262_PRRSV-1_pig	Etaarterivirus ugarco_KC787658_KRCV-2_primate	53.28%
Betaarterivirus europensis_M96262_PRRSV-1_pig	Gammaarterivirus lacdeh_U15146_LDV_rat	43.72%
Betaarterivirus europensis_M96262_PRRSV-1_pig	Iotaarterivirus debrazmo_KP126831_DeBMAV_primate	53.65%

Betaarterivirus europensis_M96262_PRRSV-1_pig	Iotaarterivirus kibreg1_JX473849_KRTGV-1_primate	54.66%
Betaarterivirus europensis_M96262_PRRSV-1_pig	Iotaarterivirus pejah_KR139839_PBJV_primate	55.42%
Betaarterivirus europensis_M96262_PRRSV-1_pig	Kappaarterivirus wobum_JN116253_WPDV_possum	69.47%
Betaarterivirus europensis_M96262_PRRSV-1_pig	Lambdaarterivirus afriporav_KP026921_APRAV_rat	58.79%
Betaarterivirus europensis_M96262_PRRSV-1_pig	Muarterivirus afrigant_MF324848_OSV-1_shrew	61.34%
Betaarterivirus europensis_M96262_PRRSV-1_pig	Muarterivirus afrigant_MF324849_OSV-2_shrew	60.82%
Betaarterivirus europensis_M96262_PRRSV-1_pig	Muarterivirus afrigant_MG264317_OSV-3_shrew	60.31%
Betaarterivirus europensis_M96262_PRRSV-1_pig	Nuarterivirus guemel_KY369969_RtClanAV_rat	36.68%
Betaarterivirus europensis_M96262_PRRSV-1_pig	Thetaarterivirus kafuba_KT447550_KKCBV_primate	51.52%
Betaarterivirus europensis_M96262_PRRSV-1_pig	Thetaarterivirus mikelba1_KM110938_MYBV-1_primate	53.54%
Betaarterivirus europensis_M96262_PRRSV-1_pig	Unclassified_KP280007_RtDs-Arterivirus -4_rat	40.70%
Betaarterivirus europensis_M96262_PRRSV-1_pig	Unclassified_MT085132_RtRe-ArtV-Tka2010B_rat	47.24%
Betaarterivirus europensis_M96262_PRRSV-1_pig	Unclassified_MT085133_RtRe-ArtV-Tka2010C_rat	47.49%
Betaarterivirus europensis_M96262_PRRSV-1_pig	Unclassified_MT085136_RtRe-ArtV-Tl2006A_rat	48.49%
Betaarterivirus europensis_M96262_PRRSV-1_pig	Unclassified_OQ715736_JSV-1_shrew	60.05%
Betaarterivirus europensis_M96262_PRRSV-1_pig	Zetaarterivirus ugarco_KC787630_KRCV-1_primate	52.14%
Betaarterivirus europensis_MT008024_PRRSV-1_pig	Betaarterivirus europensis_MT311646_PRRSV-1_pig	7.77%
Betaarterivirus europensis_MT008024_PRRSV-1_pig	Muarterivirus afrigant_MF324848_OSV-1_shrew	60.57%
Betaarterivirus europensis_MT008024_PRRSV-1_pig	Nuarterivirus guemel_KY369969_RtClanAV_rat	36.18%
Betaarterivirus europensis_MT008024_PRRSV-1_pig	Thetaarterivirus kafuba_KT447550_KKCBV_primate	51.77%
Betaarterivirus europensis_MT008024_PRRSV-1_pig	Thetaarterivirus mikelba1_KM110938_MYBV-1_primate	53.54%
Betaarterivirus europensis_MT008024_PRRSV-1_pig	Unclassified_MT085132_RtRe-ArtV-Tka2010B_rat	46.48%
Betaarterivirus europensis_MT008024_PRRSV-1_pig	Unclassified_MT085133_RtRe-ArtV-Tka2010C_rat	46.73%
Betaarterivirus europensis_MT008024_PRRSV-1_pig	Unclassified_MT085136_RtRe-ArtV-Tl2006A_rat	47.74%

Betaarterivirus europensis_MT008024_PRRSV-1_pig	Unclassified_OQ715736_JSV-1_shrew	59.54%
Betaarterivirus europensis_MT008024_PRRSV-1_pig	Zetaarterivirus ugarco_KC787630_KRCV-1_primate	52.64%
Betaarterivirus europensis_MT311646_PRRSV-1_pig	Muarterivirus afrigant_MF324848_OSV-1_shrew	61.34%
Betaarterivirus europensis_MT311646_PRRSV-1_pig	Nuarterivirus guemel_KY369969_RtClanAV_rat	36.43%
Betaarterivirus europensis_MT311646_PRRSV-1_pig	Thetaarterivirus kafuba_KT447550_KKCBV_primate	51.77%
Betaarterivirus europensis_MT311646_PRRSV-1_pig	Thetaarterivirus mikelba1_KM110938_MYBV-1_primate	53.28%
Betaarterivirus europensis_MT311646_PRRSV-1_pig	Unclassified_OQ715736_JSV-1_shrew	60.05%
Betaarterivirus europensis_MT311646_PRRSV-1_pig	Zetaarterivirus ugarco_KC787630_KRCV-1_primate	51.89%
Betaarterivirus ninrav_KU302440_RtClonAV_rat	Betaarterivirus americense_EF112445_PRRSV-2_pig	25.75%
Betaarterivirus ninrav_KU302440_RtClonAV_rat	Betaarterivirus americense_JN654459_PRRSV-2__pig	26.50%
Betaarterivirus ninrav_KU302440_RtClonAV_rat	Betaarterivirus americense_JQ308798_PRRSV-2_pig	26.00%
Betaarterivirus ninrav_KU302440_RtClonAV_rat	Betaarterivirus europensis__KX668221_PRRSV-1_pig	30.83%
Betaarterivirus ninrav_KU302440_RtClonAV_rat	Betaarterivirus europensis_JF802085_PRRSV-1_pig	31.41%
Betaarterivirus ninrav_KU302440_RtClonAV_rat	Betaarterivirus europensis_MT008024_PRRSV-1_pig	31.33%
Betaarterivirus ninrav_KU302440_RtClonAV_rat	Betaarterivirus europensis_MT311646_PRRSV-1_pig	31.58%
Betaarterivirus ninrav_KU302440_RtClonAV_rat	Betaarterivirus sheoin_KY369968_RtEiAV_rat	23.25%
Betaarterivirus ninrav_KU302440_RtClonAV_rat	Betaarterivirus timiclar_KY369967_RtMcAV_rat	25.25%
Betaarterivirus ninrav_KU302440_RtClonAV_rat	Deltaarterivirus hemfev_AF180391_SHFV_primate	53.77%
Betaarterivirus ninrav_KU302440_RtClonAV_rat	Epsilonarterivirus hemcep_KM677927_SHEV_primate	51.51%
Betaarterivirus ninrav_KU302440_RtClonAV_rat	Epsilonarterivirus safriver_KR862307_FSVV_primate	49.75%
Betaarterivirus ninrav_KU302440_RtClonAV_rat	Epsilonarterivirus zamalb_KT166441_ZMbV-1_primate	50.25%
Betaarterivirus ninrav_KU302440_RtClonAV_rat	Etaarterivirus ugarco_KC787658_KRCV-2_primate	54.41%
Betaarterivirus ninrav_KU302440_RtClonAV_rat	Gammaarterivirus lacdeh_U15146_LDV_rat	44.72%
Betaarterivirus ninrav_KU302440_RtClonAV_rat	Iotaarterivirus debrazmo_KP126831_DeBMAV_primate	54.27%

Betaarterivirus ninrav_KU302440_RtClonAV_rat	Iotaarterivirus kibreg1_JX473849_KRTGV-1_primate	53.77%
Betaarterivirus ninrav_KU302440_RtClonAV_rat	Iotaarterivirus pejah_KR139839_PBJV_primate	54.27%
Betaarterivirus ninrav_KU302440_RtClonAV_rat	Kappaarterivirus wobum_JN116253_WPDV_possum	69.80%
Betaarterivirus ninrav_KU302440_RtClonAV_rat	Lambdaarterivirus afriporav_KP026921_APRAV_rat	59.55%
Betaarterivirus ninrav_KU302440_RtClonAV_rat	Muarterivirus afrigant_MF324848_OSV-1_shrew	61.34%
Betaarterivirus ninrav_KU302440_RtClonAV_rat	Muarterivirus afrigant_MF324849_OSV-2_shrew	61.08%
Betaarterivirus ninrav_KU302440_RtClonAV_rat	Muarterivirus afrigant_MG264317_OSV-3_shrew	60.82%
Betaarterivirus ninrav_KU302440_RtClonAV_rat	Nuarterivirus guemel_KY369969_RtClanAV_rat	38.44%
Betaarterivirus ninrav_KU302440_RtClonAV_rat	Thetaarterivirus kafuba_KT447550_KKCBV_primate	54.16%
Betaarterivirus ninrav_KU302440_RtClonAV_rat	Thetaarterivirus mikelba1_KM110938_MYBV-1_primate	55.67%
Betaarterivirus ninrav_KU302440_RtClonAV_rat	Unclassified_KP280007_RtDs-Arterivirus -4_rat	40.45%
Betaarterivirus ninrav_KU302440_RtClonAV_rat	Unclassified_MT085132_RtRe-ArtV-Tka2010B_rat	48.74%
Betaarterivirus ninrav_KU302440_RtClonAV_rat	Unclassified_MT085133_RtRe-ArtV-Tka2010C_rat	48.49%
Betaarterivirus ninrav_KU302440_RtClonAV_rat	Unclassified_MT085136_RtRe-ArtV-Tl2006A_rat	47.74%
Betaarterivirus ninrav_KU302440_RtClonAV_rat	Unclassified_OQ715736_JSV-1_shrew	59.02%
Betaarterivirus ninrav_KU302440_RtClonAV_rat	Zetaarterivirus ugarco_KC787630_KRCV-1_primate	53.52%
Betaarterivirus sheoin_KY369968_RtEiAV_rat	Betaarterivirus americense_EF112445_PRRSV-2_pig	25.00%
Betaarterivirus sheoin_KY369968_RtEiAV_rat	Betaarterivirus americense_JN654459_PRRSV-2__pig	25.75%
Betaarterivirus sheoin_KY369968_RtEiAV_rat	Betaarterivirus americense_JQ308798_PRRSV-2_pig	26.00%
Betaarterivirus sheoin_KY369968_RtEiAV_rat	Betaarterivirus europensis__KX668221_PRRSV-1_pig	30.83%
Betaarterivirus sheoin_KY369968_RtEiAV_rat	Betaarterivirus europensis_JF802085_PRRSV-1_pig	31.91%
Betaarterivirus sheoin_KY369968_RtEiAV_rat	Betaarterivirus europensis_MT008024_PRRSV-1_pig	30.83%
Betaarterivirus sheoin_KY369968_RtEiAV_rat	Betaarterivirus europensis_MT311646_PRRSV-1_pig	30.83%
Betaarterivirus sheoin_KY369968_RtEiAV_rat	Betaarterivirus timiclar_KY369967_RtMcAV_rat	27.50%

Betaarterivirus sheoin_KY369968_RtEiAV_rat	Deltaarterivirus hemfev_AF180391_SHFV_primate	53.52%
Betaarterivirus sheoin_KY369968_RtEiAV_rat	Epsilonarterivirus hemcep_KM677927_SHEV_primate	52.26%
Betaarterivirus sheoin_KY369968_RtEiAV_rat	Epsilonarterivirus safriver_KR862307_FSVV_primate	50.50%
Betaarterivirus sheoin_KY369968_RtEiAV_rat	Epsilonarterivirus zamalb_KT166441_ZMbV-1_primate	51.01%
Betaarterivirus sheoin_KY369968_RtEiAV_rat	Etaarterivirus ugarco_KC787658_KRCV-2_primate	54.91%
Betaarterivirus sheoin_KY369968_RtEiAV_rat	Gammaarterivirus lacdeh_U15146_LDV_rat	46.98%
Betaarterivirus sheoin_KY369968_RtEiAV_rat	Iotaarterivirus debrazmo_KP126831_DeBMAV_primate	54.77%
Betaarterivirus sheoin_KY369968_RtEiAV_rat	Iotaarterivirus kibreg1_JX473849_KRTGV-1_primate	55.28%
Betaarterivirus sheoin_KY369968_RtEiAV_rat	Iotaarterivirus pejah_KR139839_PBJV_primate	56.78%
Betaarterivirus sheoin_KY369968_RtEiAV_rat	Kappaarterivirus wobum_JN116253_WPDV_possum	70.05%
Betaarterivirus sheoin_KY369968_RtEiAV_rat	Lambdaarterivirus afriporav_KP026921_APRAV_rat	59.55%
Betaarterivirus sheoin_KY369968_RtEiAV_rat	Muarterivirus afrigant_MF324848_OSV-1_shrew	62.11%
Betaarterivirus sheoin_KY369968_RtEiAV_rat	Muarterivirus afrigant_MF324849_OSV-2_shrew	61.86%
Betaarterivirus sheoin_KY369968_RtEiAV_rat	Muarterivirus afrigant_MG264317_OSV-3_shrew	61.60%
Betaarterivirus sheoin_KY369968_RtEiAV_rat	Nuarterivirus guemel_KY369969_RtClanAV_rat	35.43%
Betaarterivirus sheoin_KY369968_RtEiAV_rat	Thetaarterivirus kafuba_KT447550_KKCBV_primate	53.65%
Betaarterivirus sheoin_KY369968_RtEiAV_rat	Thetaarterivirus mikelba1_KM110938_MYBV-1_primate	55.16%
Betaarterivirus sheoin_KY369968_RtEiAV_rat	Unclassified_KP280007_RtDs-Arterivirus -4_rat	43.47%
Betaarterivirus sheoin_KY369968_RtEiAV_rat	Unclassified_MT085132_RtRe-ArtV-Tka2010B_rat	50.00%
Betaarterivirus sheoin_KY369968_RtEiAV_rat	Unclassified_MT085133_RtRe-ArtV-Tka2010C_rat	50.00%
Betaarterivirus sheoin_KY369968_RtEiAV_rat	Unclassified_MT085136_RtRe-ArtV-Tl2006A_rat	49.50%
Betaarterivirus sheoin_KY369968_RtEiAV_rat	Unclassified_OQ715736_JSV-1_shrew	60.57%
Betaarterivirus sheoin_KY369968_RtEiAV_rat	Zetaarterivirus ugarco_KC787630_KRCV-1_primate	53.77%
Betaarterivirus timiclar_KY369967_RtMcAV_rat	Betaarterivirus americense_EF112445_PRRSV-2_pig	25.50%

Betaarterivirus timiclar_KY369967_RtMcAV_rat	Betaarterivirus americense_JN654459_PRRSV-2__pig	26.25%
Betaarterivirus timiclar_KY369967_RtMcAV_rat	Betaarterivirus americense_JQ308798_PRRSV-2_pig	25.25%
Betaarterivirus timiclar_KY369967_RtMcAV_rat	Betaarterivirus europensis__KX668221_PRRSV-1_pig	33.08%
Betaarterivirus timiclar_KY369967_RtMcAV_rat	Betaarterivirus europensis_JF802085_PRRSV-1_pig	35.18%
Betaarterivirus timiclar_KY369967_RtMcAV_rat	Betaarterivirus europensis_MT008024_PRRSV-1_pig	31.83%
Betaarterivirus timiclar_KY369967_RtMcAV_rat	Betaarterivirus europensis_MT311646_PRRSV-1_pig	31.58%
Betaarterivirus timiclar_KY369967_RtMcAV_rat	Deltaarterivirus hemfev_AF180391_SHFV_primate	54.52%
Betaarterivirus timiclar_KY369967_RtMcAV_rat	Epsilonarterivirus hemcep_KM677927_SHEV_primate	53.27%
Betaarterivirus timiclar_KY369967_RtMcAV_rat	Epsilonarterivirus safriver_KR862307_FSVV_primate	52.01%
Betaarterivirus timiclar_KY369967_RtMcAV_rat	Epsilonarterivirus zamalb_KT166441_ZMbV-1_primate	51.26%
Betaarterivirus timiclar_KY369967_RtMcAV_rat	Etaarterivirus ugarco_KC787658_KRCV-2_primate	54.16%
Betaarterivirus timiclar_KY369967_RtMcAV_rat	Gammaarterivirus lacdeh_U15146_LDV_rat	44.22%
Betaarterivirus timiclar_KY369967_RtMcAV_rat	Iotaarterivirus debrazmo_KP126831_DeBMAV_primate	54.27%
Betaarterivirus timiclar_KY369967_RtMcAV_rat	Iotaarterivirus kibreg1_JX473849_KRTGV-1_primate	55.03%
Betaarterivirus timiclar_KY369967_RtMcAV_rat	Iotaarterivirus pejah_KR139839_PBJV_primate	54.52%
Betaarterivirus timiclar_KY369967_RtMcAV_rat	Kappaarterivirus wobum_JN116253_WPDV_possum	69.80%
Betaarterivirus timiclar_KY369967_RtMcAV_rat	Lambdaarterivirus afriporav_KP026921_APRAV_rat	56.53%
Betaarterivirus timiclar_KY369967_RtMcAV_rat	Muarterivirus afrigant_MF324848_OSV-1_shrew	61.34%
Betaarterivirus timiclar_KY369967_RtMcAV_rat	Muarterivirus afrigant_MF324849_OSV-2_shrew	61.08%
Betaarterivirus timiclar_KY369967_RtMcAV_rat	Muarterivirus afrigant_MG264317_OSV-3_shrew	60.31%
Betaarterivirus timiclar_KY369967_RtMcAV_rat	Nuarterivirus guemel_KY369969_RtClanAV_rat	37.44%
Betaarterivirus timiclar_KY369967_RtMcAV_rat	Thetaarterivirus kafuba_KT447550_KKCBV_primate	54.66%
Betaarterivirus timiclar_KY369967_RtMcAV_rat	Thetaarterivirus mikelba1_KM110938_MYBV-1_primate	54.91%
Betaarterivirus timiclar_KY369967_RtMcAV_rat	Unclassified_KP280007_RtDs-Arterivirus -4_rat	42.96%

Betaarterivirus timiclar_KY369967_RtMcAV_rat	Unclassified_MT085132_RtRe-ArtV-Tka2010B_rat	48.74%
Betaarterivirus timiclar_KY369967_RtMcAV_rat	Unclassified_MT085133_RtRe-ArtV-Tka2010C_rat	48.49%
Betaarterivirus timiclar_KY369967_RtMcAV_rat	Unclassified_MT085136_RtRe-ArtV-Tl2006A_rat	46.98%
Betaarterivirus timiclar_KY369967_RtMcAV_rat	Unclassified_OQ715736_JSV-1_shrew	59.79%
Betaarterivirus timiclar_KY369967_RtMcAV_rat	Zetaarterivirus ugarco_KC787630_KRCV-1_primate	53.27%
Deltaarterivirus hemfev_AF180391_SHFV_primate	Betaarterivirus americense_EF112445_PRRSV-2_pig	53.52%
Deltaarterivirus hemfev_AF180391_SHFV_primate	Betaarterivirus americense_JN654459_PRRSV-2__pig	52.76%
Deltaarterivirus hemfev_AF180391_SHFV_primate	Betaarterivirus americense_JQ308798_PRRSV-2_pig	53.77%
Deltaarterivirus hemfev_AF180391_SHFV_primate	Betaarterivirus europensis__KX668221_PRRSV-1_pig	51.89%
Deltaarterivirus hemfev_AF180391_SHFV_primate	Betaarterivirus europensis_JF802085_PRRSV-1_pig	53.03%
Deltaarterivirus hemfev_AF180391_SHFV_primate	Betaarterivirus europensis_MT008024_PRRSV-1_pig	53.15%
Deltaarterivirus hemfev_AF180391_SHFV_primate	Betaarterivirus europensis_MT311646_PRRSV-1_pig	52.90%
Deltaarterivirus hemfev_AF180391_SHFV_primate	Epsilonarterivirus hemcep_KM677927_SHEV_primate	45.61%
Deltaarterivirus hemfev_AF180391_SHFV_primate	Epsilonarterivirus safriver_KR862307_FSVV_primate	46.37%
Deltaarterivirus hemfev_AF180391_SHFV_primate	Epsilonarterivirus zamalb_KT166441_ZMbV-1_primate	45.61%
Deltaarterivirus hemfev_AF180391_SHFV_primate	Etaarterivirus ugarco_KC787658_KRCV-2_primate	42.11%
Deltaarterivirus hemfev_AF180391_SHFV_primate	Gammaarterivirus lacdeh_U15146_LDV_rat	54.29%
Deltaarterivirus hemfev_AF180391_SHFV_primate	Iotaarterivirus debrazmo_KP126831_DeBMAV_primate	36.00%
Deltaarterivirus hemfev_AF180391_SHFV_primate	Iotaarterivirus kibreg1_JX473849_KRTGV-1_primate	36.75%
Deltaarterivirus hemfev_AF180391_SHFV_primate	Iotaarterivirus pejah_KR139839_PBJV_primate	34.25%
Deltaarterivirus hemfev_AF180391_SHFV_primate	Kappaarterivirus wobum_JN116253_WPDV_possum	68.45%
Deltaarterivirus hemfev_AF180391_SHFV_primate	Lambdaarterivirus afriporav_KP026921_APRAV_rat	58.29%
Deltaarterivirus hemfev_AF180391_SHFV_primate	Muarterivirus afrigant_MF324848_OSV-1_shrew	64.08%
Deltaarterivirus hemfev_AF180391_SHFV_primate	Muarterivirus afrigant_MF324849_OSV-2_shrew	63.57%

Deltaarterivirus hemfev_AF180391_SHFV_primate	Muarterivirus afrigant_MG264317_OSV-3_shrew	63.31%
Deltaarterivirus hemfev_AF180391_SHFV_primate	Nuarterivirus guemel_KY369969_RtClanAV_rat	51.01%
Deltaarterivirus hemfev_AF180391_SHFV_primate	Thetaarterivirus kafuba_KT447550_KKCBV_primate	34.84%
Deltaarterivirus hemfev_AF180391_SHFV_primate	Thetaarterivirus mikelba1_KM110938_MYBV-1_primate	37.09%
Deltaarterivirus hemfev_AF180391_SHFV_primate	Unclassified_KP280007_RtDs-Arterivirus -4_rat	53.54%
Deltaarterivirus hemfev_AF180391_SHFV_primate	Unclassified_MT085132_RtRe-ArtV-Tka2010B_rat	58.33%
Deltaarterivirus hemfev_AF180391_SHFV_primate	Unclassified_MT085133_RtRe-ArtV-Tka2010C_rat	58.33%
Deltaarterivirus hemfev_AF180391_SHFV_primate	Unclassified_MT085136_RtRe-ArtV-Tl2006A_rat	57.83%
Deltaarterivirus hemfev_AF180391_SHFV_primate	Unclassified_OQ715736_JSV-1_shrew	62.27%
Deltaarterivirus hemfev_AF180391_SHFV_primate	Zetaarterivirus ugarco_KC787630_KRCV-1_primate	44.36%
Epsilonarterivirus hemcep_KM677927_SHEV_primate	Betaarterivirus americense_JN654459_PRRSV-2__pig	51.26%
Epsilonarterivirus hemcep_KM677927_SHEV_primate	Betaarterivirus americense_JQ308798_PRRSV-2_pig	52.26%
Epsilonarterivirus hemcep_KM677927_SHEV_primate	Betaarterivirus europensis__KX668221_PRRSV-1_pig	50.13%
Epsilonarterivirus hemcep_KM677927_SHEV_primate	Betaarterivirus europensis_JF802085_PRRSV-1_pig	52.02%
Epsilonarterivirus hemcep_KM677927_SHEV_primate	Betaarterivirus europensis_MT008024_PRRSV-1_pig	52.14%
Epsilonarterivirus hemcep_KM677927_SHEV_primate	Betaarterivirus europensis_MT311646_PRRSV-1_pig	51.89%
Epsilonarterivirus hemcep_KM677927_SHEV_primate	Epsilonarterivirus safriver_KR862307_FSVV_primate	18.66%
Epsilonarterivirus hemcep_KM677927_SHEV_primate	Epsilonarterivirus zamalb_KT166441_ZMbV-1_primate	20.15%
Epsilonarterivirus hemcep_KM677927_SHEV_primate	Etaarterivirus ugarco_KC787658_KRCV-2_primate	43.25%
Epsilonarterivirus hemcep_KM677927_SHEV_primate	Gammaarterivirus lacdeh_U15146_LDV_rat	54.80%
Epsilonarterivirus hemcep_KM677927_SHEV_primate	Iotaarterivirus debrazmo_KP126831_DeBMAV_primate	45.86%
Epsilonarterivirus hemcep_KM677927_SHEV_primate	Iotaarterivirus kibreg1_JX473849_KRTGV-1_primate	46.62%
Epsilonarterivirus hemcep_KM677927_SHEV_primate	Iotaarterivirus pejah_KR139839_PBJV_primate	43.61%
Epsilonarterivirus hemcep_KM677927_SHEV_primate	Kappaarterivirus wobum_JN116253_WPDV_possum	69.04%

Epsilonarterivirus hemcep_KM677927_SHEV_primate	Lambdaarterivirus afriporav_KP026921_APRAV_rat	57.50%
Epsilonarterivirus hemcep_KM677927_SHEV_primate	Muarterivirus afrigant_MF324848_OSV-1_shrew	61.18%
Epsilonarterivirus hemcep_KM677927_SHEV_primate	Muarterivirus afrigant_MF324849_OSV-2_shrew	60.93%
Epsilonarterivirus hemcep_KM677927_SHEV_primate	Muarterivirus afrigant_MG264317_OSV-3_shrew	60.41%
Epsilonarterivirus hemcep_KM677927_SHEV_primate	Nuarterivirus guemel_KY369969_RtClanAV_rat	50.25%
Epsilonarterivirus hemcep_KM677927_SHEV_primate	Thetaarterivirus kafuba_KT447550_KKCBV_primate	44.61%
Epsilonarterivirus hemcep_KM677927_SHEV_primate	Thetaarterivirus mikelba1_KM110938_MYBV-1_primate	45.61%
Epsilonarterivirus hemcep_KM677927_SHEV_primate	Unclassified_KP280007_RtDs-Arterivirus -4_rat	52.27%
Epsilonarterivirus hemcep_KM677927_SHEV_primate	Unclassified_MT085132_RtRe-ArtV-Tka2010B_rat	55.56%
Epsilonarterivirus hemcep_KM677927_SHEV_primate	Unclassified_MT085133_RtRe-ArtV-Tka2010C_rat	55.56%
Epsilonarterivirus hemcep_KM677927_SHEV_primate	Unclassified_MT085136_RtRe-ArtV-Tl2006A_rat	55.56%
Epsilonarterivirus hemcep_KM677927_SHEV_primate	Unclassified_OQ715736_JSV-1_shrew	60.15%
Epsilonarterivirus hemcep_KM677927_SHEV_primate	Zetaarterivirus ugarco_KC787630_KRCV-1_primate	46.63%
Epsilonarterivirus safriver_KR862307_FSVV_primate	Betaarterivirus americense_JN654459_PRRSV-2__pig	50.00%
Epsilonarterivirus safriver_KR862307_FSVV_primate	Betaarterivirus americense_JQ308798_PRRSV-2_pig	51.01%
Epsilonarterivirus safriver_KR862307_FSVV_primate	Betaarterivirus europensis__KX668221_PRRSV-1_pig	49.87%
Epsilonarterivirus safriver_KR862307_FSVV_primate	Betaarterivirus europensis_JF802085_PRRSV-1_pig	50.51%
Epsilonarterivirus safriver_KR862307_FSVV_primate	Betaarterivirus europensis_MT008024_PRRSV-1_pig	50.88%
Epsilonarterivirus safriver_KR862307_FSVV_primate	Betaarterivirus europensis_MT311646_PRRSV-1_pig	50.63%
Epsilonarterivirus safriver_KR862307_FSVV_primate	Epsilonarterivirus zamalb_KT166441_ZMbV-1_primate	17.41%
Epsilonarterivirus safriver_KR862307_FSVV_primate	Etaarterivirus ugarco_KC787658_KRCV-2_primate	44.25%
Epsilonarterivirus safriver_KR862307_FSVV_primate	Gammaarterivirus lacdeh_U15146_LDV_rat	53.54%
Epsilonarterivirus safriver_KR862307_FSVV_primate	Iotaarterivirus debrazmo_KP126831_DeBMAV_primate	43.86%
Epsilonarterivirus safriver_KR862307_FSVV_primate	Iotaarterivirus kibreg1_JX473849_KRTGV-1_primate	46.37%

Epsilonarterivirus safriver_KR862307_FSVV_primate	Iotaarterivirus pejah_KR139839_PBJV_primate	39.10%
Epsilonarterivirus safriver_KR862307_FSVV_primate	Kappaarterivirus wobum_JN116253_WPDV_possum	70.30%
Epsilonarterivirus safriver_KR862307_FSVV_primate	Lambdaarterivirus afriporav_KP026921_APRAV_rat	56.50%
Epsilonarterivirus safriver_KR862307_FSVV_primate	Muarterivirus afrigant_MF324848_OSV-1_shrew	62.98%
Epsilonarterivirus safriver_KR862307_FSVV_primate	Muarterivirus afrigant_MF324849_OSV-2_shrew	62.72%
Epsilonarterivirus safriver_KR862307_FSVV_primate	Muarterivirus afrigant_MG264317_OSV-3_shrew	62.47%
Epsilonarterivirus safriver_KR862307_FSVV_primate	Nuarterivirus guemel_KY369969_RtClanAV_rat	48.48%
Epsilonarterivirus safriver_KR862307_FSVV_primate	Thetaarterivirus kafuba_KT447550_KKCBV_primate	44.61%
Epsilonarterivirus safriver_KR862307_FSVV_primate	Thetaarterivirus mikelba1_KM110938_MYBV-1_primate	47.37%
Epsilonarterivirus safriver_KR862307_FSVV_primate	Unclassified_KP280007_RtDs-Arterivirus -4_rat	51.26%
Epsilonarterivirus safriver_KR862307_FSVV_primate	Unclassified_MT085132_RtRe-ArtV-Tka2010B_rat	54.04%
Epsilonarterivirus safriver_KR862307_FSVV_primate	Unclassified_MT085133_RtRe-ArtV-Tka2010C_rat	53.79%
Epsilonarterivirus safriver_KR862307_FSVV_primate	Unclassified_MT085136_RtRe-ArtV-Tl2006A_rat	54.04%
Epsilonarterivirus safriver_KR862307_FSVV_primate	Unclassified_OQ715736_JSV-1_shrew	61.44%
Epsilonarterivirus safriver_KR862307_FSVV_primate	Zetaarterivirus ugarco_KC787630_KRCV-1_primate	44.64%
Epsilonarterivirus zamalb_KT166441_ZMbV-1_primate	Betaarterivirus americense_JN654459_PRRSV-2__pig	49.25%
Epsilonarterivirus zamalb_KT166441_ZMbV-1_primate	Betaarterivirus americense_JQ308798_PRRSV-2_pig	50.00%
Epsilonarterivirus zamalb_KT166441_ZMbV-1_primate	Betaarterivirus europensis__KX668221_PRRSV-1_pig	51.13%
Epsilonarterivirus zamalb_KT166441_ZMbV-1_primate	Betaarterivirus europensis_JF802085_PRRSV-1_pig	51.52%
Epsilonarterivirus zamalb_KT166441_ZMbV-1_primate	Betaarterivirus europensis_MT008024_PRRSV-1_pig	51.13%
Epsilonarterivirus zamalb_KT166441_ZMbV-1_primate	Betaarterivirus europensis_MT311646_PRRSV-1_pig	51.39%
Epsilonarterivirus zamalb_KT166441_ZMbV-1_primate	Etaarterivirus ugarco_KC787658_KRCV-2_primate	43.50%
Epsilonarterivirus zamalb_KT166441_ZMbV-1_primate	Gammaarterivirus lacdeh_U15146_LDV_rat	53.79%
Epsilonarterivirus zamalb_KT166441_ZMbV-1_primate	Iotaarterivirus debrazmo_KP126831_DeBMAV_primate	42.86%

Epsilonarterivirus zamalb_KT166441_ZMbV-1_primate	Iotaarterivirus kibreg1_JX473849_KRTGV-1_primate	45.86%
Epsilonarterivirus zamalb_KT166441_ZMbV-1_primate	Iotaarterivirus pejah_KR139839_PBJV_primate	43.11%
Epsilonarterivirus zamalb_KT166441_ZMbV-1_primate	Kappaarterivirus wobum_JN116253_WPDV_possum	69.54%
Epsilonarterivirus zamalb_KT166441_ZMbV-1_primate	Lambdaarterivirus afriporav_KP026921_APRAV_rat	56.50%
Epsilonarterivirus zamalb_KT166441_ZMbV-1_primate	Muarterivirus afrigant_MF324848_OSV-1_shrew	62.21%
Epsilonarterivirus zamalb_KT166441_ZMbV-1_primate	Muarterivirus afrigant_MF324849_OSV-2_shrew	62.21%
Epsilonarterivirus zamalb_KT166441_ZMbV-1_primate	Muarterivirus afrigant_MG264317_OSV-3_shrew	62.21%
Epsilonarterivirus zamalb_KT166441_ZMbV-1_primate	Nuarterivirus guemel_KY369969_RtClanAV_rat	50.51%
Epsilonarterivirus zamalb_KT166441_ZMbV-1_primate	Thetaarterivirus kafuba_KT447550_KKCBV_primate	44.86%
Epsilonarterivirus zamalb_KT166441_ZMbV-1_primate	Thetaarterivirus mikelba1_KM110938_MYBV-1_primate	45.11%
Epsilonarterivirus zamalb_KT166441_ZMbV-1_primate	Unclassified_KP280007_RtDs-Arterivirus -4_rat	53.79%
Epsilonarterivirus zamalb_KT166441_ZMbV-1_primate	Unclassified_MT085132_RtRe-ArtV-Tka2010B_rat	55.30%
Epsilonarterivirus zamalb_KT166441_ZMbV-1_primate	Unclassified_MT085133_RtRe-ArtV-Tka2010C_rat	55.05%
Epsilonarterivirus zamalb_KT166441_ZMbV-1_primate	Unclassified_MT085136_RtRe-ArtV-Tl2006A_rat	55.56%
Epsilonarterivirus zamalb_KT166441_ZMbV-1_primate	Unclassified_OQ715736_JSV-1_shrew	60.67%
Epsilonarterivirus zamalb_KT166441_ZMbV-1_primate	Zetaarterivirus ugarco_KC787630_KRCV-1_primate	46.63%
Etaarterivirus ugarco_KC787658_KRCV-2_primate	Betaarterivirus americense_JN654459_PRRSV-2__pig	53.65%
Etaarterivirus ugarco_KC787658_KRCV-2_primate	Betaarterivirus americense_JQ308798_PRRSV-2_pig	54.91%
Etaarterivirus ugarco_KC787658_KRCV-2_primate	Betaarterivirus europensis__KX668221_PRRSV-1_pig	54.04%
Etaarterivirus ugarco_KC787658_KRCV-2_primate	Betaarterivirus europensis_JF802085_PRRSV-1_pig	54.94%
Etaarterivirus ugarco_KC787658_KRCV-2_primate	Betaarterivirus europensis_MT008024_PRRSV-1_pig	52.53%
Etaarterivirus ugarco_KC787658_KRCV-2_primate	Betaarterivirus europensis_MT311646_PRRSV-1_pig	53.54%
Etaarterivirus ugarco_KC787658_KRCV-2_primate	Gammaarterivirus lacdeh_U15146_LDV_rat	56.46%
Etaarterivirus ugarco_KC787658_KRCV-2_primate	Iotaarterivirus debrazmo_KP126831_DeBMAV_primate	42.86%

Etaarterivirus ugarco_KC787658_KRCV-2_primate	Iotaarterivirus kibreg1_JX473849_KRTGV-1_primate	41.85%
Etaarterivirus ugarco_KC787658_KRCV-2_primate	Iotaarterivirus pejah_KR139839_PBJV_primate	42.61%
Etaarterivirus ugarco_KC787658_KRCV-2_primate	Kappaarterivirus wobum_JN116253_WPDV_possum	69.97%
Etaarterivirus ugarco_KC787658_KRCV-2_primate	Lambdaarterivirus afriporav_KP026921_APRAV_rat	58.54%
Etaarterivirus ugarco_KC787658_KRCV-2_primate	Muarterivirus afrigant_MF324848_OSV-1_shrew	63.05%
Etaarterivirus ugarco_KC787658_KRCV-2_primate	Muarterivirus afrigant_MF324849_OSV-2_shrew	62.27%
Etaarterivirus ugarco_KC787658_KRCV-2_primate	Muarterivirus afrigant_MG264317_OSV-3_shrew	61.76%
Etaarterivirus ugarco_KC787658_KRCV-2_primate	Nuarterivirus guemel_KY369969_RtClanAV_rat	53.42%
Etaarterivirus ugarco_KC787658_KRCV-2_primate	Thetaarterivirus kafuba_KT447550_KKCBV_primate	41.35%
Etaarterivirus ugarco_KC787658_KRCV-2_primate	Thetaarterivirus mikelba1_KM110938_MYBV-1_primate	42.61%
Etaarterivirus ugarco_KC787658_KRCV-2_primate	Unclassified_KP280007_RtDs-Arterivirus -4_rat	54.68%
Etaarterivirus ugarco_KC787658_KRCV-2_primate	Unclassified_MT085132_RtRe-ArtV-Tka2010B_rat	56.20%
Etaarterivirus ugarco_KC787658_KRCV-2_primate	Unclassified_MT085133_RtRe-ArtV-Tka2010C_rat	55.95%
Etaarterivirus ugarco_KC787658_KRCV-2_primate	Unclassified_MT085136_RtRe-ArtV-Tl2006A_rat	56.46%
Etaarterivirus ugarco_KC787658_KRCV-2_primate	Unclassified_OQ715736_JSV-1_shrew	59.17%
Etaarterivirus ugarco_KC787658_KRCV-2_primate	Zetaarterivirus ugarco_KC787630_KRCV-1_primate	43.75%
Gammaarterivirus lacdeh_U15146_LDV_rat	Betaarterivirus americense_JN654459_PRRSV-2__pig	46.48%
Gammaarterivirus lacdeh_U15146_LDV_rat	Betaarterivirus americense_JQ308798_PRRSV-2_pig	45.23%
Gammaarterivirus lacdeh_U15146_LDV_rat	Betaarterivirus europensis__KX668221_PRRSV-1_pig	44.72%
Gammaarterivirus lacdeh_U15146_LDV_rat	Betaarterivirus europensis_JF802085_PRRSV-1_pig	44.33%
Gammaarterivirus lacdeh_U15146_LDV_rat	Betaarterivirus europensis_MT008024_PRRSV-1_pig	44.47%
Gammaarterivirus lacdeh_U15146_LDV_rat	Betaarterivirus europensis_MT311646_PRRSV-1_pig	43.72%
Gammaarterivirus lacdeh_U15146_LDV_rat	Iotaarterivirus debrazmo_KP126831_DeBMAV_primate	54.29%
Gammaarterivirus lacdeh_U15146_LDV_rat	Iotaarterivirus kibreg1_JX473849_KRTGV-1_primate	56.31%

Gammaarterivirus lacdeh_U15146_LDV_rat	Iotaarterivirus pejah_KR139839_PBJV_primate	58.33%
Gammaarterivirus lacdeh_U15146_LDV_rat	Kappaarterivirus wobum_JN116253_WPDV_possum	70.92%
Gammaarterivirus lacdeh_U15146_LDV_rat	Lambdaarterivirus afriporav_KP026921_APRAV_rat	60.71%
Gammaarterivirus lacdeh_U15146_LDV_rat	Muarterivirus afrigant_MF324848_OSV-1_shrew	60.31%
Gammaarterivirus lacdeh_U15146_LDV_rat	Muarterivirus afrigant_MF324849_OSV-2_shrew	60.05%
Gammaarterivirus lacdeh_U15146_LDV_rat	Muarterivirus afrigant_MG264317_OSV-3_shrew	60.31%
Gammaarterivirus lacdeh_U15146_LDV_rat	Nuarterivirus guemel_KY369969_RtClanAV_rat	43.47%
Gammaarterivirus lacdeh_U15146_LDV_rat	Thetaarterivirus kafuba_KT447550_KKCBV_primate	54.94%
Gammaarterivirus lacdeh_U15146_LDV_rat	Thetaarterivirus mikelba1_KM110938_MYBV-1_primate	56.20%
Gammaarterivirus lacdeh_U15146_LDV_rat	Unclassified_KP280007_RtDs-Arterivirus -4_rat	41.21%
Gammaarterivirus lacdeh_U15146_LDV_rat	Unclassified_MT085132_RtRe-ArtV-Tka2010B_rat	42.46%
Gammaarterivirus lacdeh_U15146_LDV_rat	Unclassified_MT085133_RtRe-ArtV-Tka2010C_rat	41.71%
Gammaarterivirus lacdeh_U15146_LDV_rat	Unclassified_MT085136_RtRe-ArtV-Tl2006A_rat	42.46%
Gammaarterivirus lacdeh_U15146_LDV_rat	Unclassified_OQ715736_JSV-1_shrew	60.05%
Gammaarterivirus lacdeh_U15146_LDV_rat	Zetaarterivirus ugarco_KC787630_KRCV-1_primate	57.32%
Iotaarterivirus debrazmo_KP126831_DeBMAV_primate	Betaarterivirus americense_JN654459_PRRSV-2__pig	56.03%
Iotaarterivirus debrazmo_KP126831_DeBMAV_primate	Betaarterivirus americense_JQ308798_PRRSV-2_pig	56.53%
Iotaarterivirus debrazmo_KP126831_DeBMAV_primate	Betaarterivirus europensis__KX668221_PRRSV-1_pig	53.15%
Iotaarterivirus debrazmo_KP126831_DeBMAV_primate	Betaarterivirus europensis_JF802085_PRRSV-1_pig	53.03%
Iotaarterivirus debrazmo_KP126831_DeBMAV_primate	Betaarterivirus europensis_MT008024_PRRSV-1_pig	53.15%
Iotaarterivirus debrazmo_KP126831_DeBMAV_primate	Betaarterivirus europensis_MT311646_PRRSV-1_pig	53.40%
Iotaarterivirus debrazmo_KP126831_DeBMAV_primate	Iotaarterivirus kibreg1_JX473849_KRTGV-1_primate	31.50%
Iotaarterivirus debrazmo_KP126831_DeBMAV_primate	Iotaarterivirus pejah_KR139839_PBJV_primate	35.00%
Iotaarterivirus debrazmo_KP126831_DeBMAV_primate	Kappaarterivirus wobum_JN116253_WPDV_possum	68.70%

Iotaarterivirus debrazmo_KP126831_DeBMAV_primate	Lambdaarterivirus afriporav_KP026921_APRAV_rat	57.79%
Iotaarterivirus debrazmo_KP126831_DeBMAV_primate	Muarterivirus afrigant_MF324848_OSV-1_shrew	59.69%
Iotaarterivirus debrazmo_KP126831_DeBMAV_primate	Muarterivirus afrigant_MF324849_OSV-2_shrew	59.43%
Iotaarterivirus debrazmo_KP126831_DeBMAV_primate	Muarterivirus afrigant_MG264317_OSV-3_shrew	60.21%
Iotaarterivirus debrazmo_KP126831_DeBMAV_primate	Nuarterivirus guemel_KY369969_RtClanAV_rat	55.30%
Iotaarterivirus debrazmo_KP126831_DeBMAV_primate	Thetaarterivirus kafuba_KT447550_KKCBV_primate	36.59%
Iotaarterivirus debrazmo_KP126831_DeBMAV_primate	Thetaarterivirus mikelba1_KM110938_MYBV-1_primate	38.10%
Iotaarterivirus debrazmo_KP126831_DeBMAV_primate	Unclassified_KP280007_RtDs-Arterivirus -4_rat	54.55%
Iotaarterivirus debrazmo_KP126831_DeBMAV_primate	Unclassified_MT085132_RtRe-ArtV-Tka2010B_rat	56.82%
Iotaarterivirus debrazmo_KP126831_DeBMAV_primate	Unclassified_MT085133_RtRe-ArtV-Tka2010C_rat	56.57%
Iotaarterivirus debrazmo_KP126831_DeBMAV_primate	Unclassified_MT085136_RtRe-ArtV-Tl2006A_rat	56.57%
Iotaarterivirus debrazmo_KP126831_DeBMAV_primate	Unclassified_OQ715736_JSV-1_shrew	59.43%
Iotaarterivirus debrazmo_KP126831_DeBMAV_primate	Zetaarterivirus ugarco_KC787630_KRCV-1_primate	44.36%
Iotaarterivirus kibreg1_JX473849_KRTGV-1_primate	Betaarterivirus americense_JN654459_PRRSV-2__pig	55.53%
Iotaarterivirus kibreg1_JX473849_KRTGV-1_primate	Betaarterivirus americense_JQ308798_PRRSV-2_pig	56.53%
Iotaarterivirus kibreg1_JX473849_KRTGV-1_primate	Betaarterivirus europensis__KX668221_PRRSV-1_pig	54.16%
Iotaarterivirus kibreg1_JX473849_KRTGV-1_primate	Betaarterivirus europensis_JF802085_PRRSV-1_pig	54.80%
Iotaarterivirus kibreg1_JX473849_KRTGV-1_primate	Betaarterivirus europensis_MT008024_PRRSV-1_pig	53.90%
Iotaarterivirus kibreg1_JX473849_KRTGV-1_primate	Betaarterivirus europensis_MT311646_PRRSV-1_pig	54.91%
Iotaarterivirus kibreg1_JX473849_KRTGV-1_primate	Iotaarterivirus pejah_KR139839_PBJV_primate	36.50%
Iotaarterivirus kibreg1_JX473849_KRTGV-1_primate	Kappaarterivirus wobum_JN116253_WPDV_possum	68.96%
Iotaarterivirus kibreg1_JX473849_KRTGV-1_primate	Lambdaarterivirus afriporav_KP026921_APRAV_rat	60.05%
Iotaarterivirus kibreg1_JX473849_KRTGV-1_primate	Muarterivirus afrigant_MF324848_OSV-1_shrew	61.76%
Iotaarterivirus kibreg1_JX473849_KRTGV-1_primate	Muarterivirus afrigant_MF324849_OSV-2_shrew	61.50%

Iotaarterivirus kibreg1_JX473849_KRTGV-1_primate	Muarterivirus afrigant_MG264317_OSV-3_shrew	60.98%
Iotaarterivirus kibreg1_JX473849_KRTGV-1_primate	Nuarterivirus guemel_KY369969_RtClanAV_rat	55.05%
Iotaarterivirus kibreg1_JX473849_KRTGV-1_primate	Thetaarterivirus kafuba_KT447550_KKCBV_primate	33.58%
Iotaarterivirus kibreg1_JX473849_KRTGV-1_primate	Thetaarterivirus mikelba1_KM110938_MYBV-1_primate	35.59%
Iotaarterivirus kibreg1_JX473849_KRTGV-1_primate	Unclassified_KP280007_RtDs-Arterivirus -4_rat	53.54%
Iotaarterivirus kibreg1_JX473849_KRTGV-1_primate	Unclassified_MT085132_RtRe-ArtV-Tka2010B_rat	56.31%
Iotaarterivirus kibreg1_JX473849_KRTGV-1_primate	Unclassified_MT085133_RtRe-ArtV-Tka2010C_rat	56.06%
Iotaarterivirus kibreg1_JX473849_KRTGV-1_primate	Unclassified_MT085136_RtRe-ArtV-Tl2006A_rat	56.06%
Iotaarterivirus kibreg1_JX473849_KRTGV-1_primate	Unclassified_OQ715736_JSV-1_shrew	61.50%
Iotaarterivirus kibreg1_JX473849_KRTGV-1_primate	Zetaarterivirus ugarco_KC787630_KRCV-1_primate	45.36%
Iotaarterivirus pejah_KR139839_PBJV_primate	Betaarterivirus americense_JN654459_PRRSV-2__pig	55.53%
Iotaarterivirus pejah_KR139839_PBJV_primate	Betaarterivirus americense_JQ308798_PRRSV-2_pig	56.03%
Iotaarterivirus pejah_KR139839_PBJV_primate	Betaarterivirus europensis__KX668221_PRRSV-1_pig	55.42%
Iotaarterivirus pejah_KR139839_PBJV_primate	Betaarterivirus europensis_JF802085_PRRSV-1_pig	56.57%
Iotaarterivirus pejah_KR139839_PBJV_primate	Betaarterivirus europensis_MT008024_PRRSV-1_pig	55.67%
Iotaarterivirus pejah_KR139839_PBJV_primate	Betaarterivirus europensis_MT311646_PRRSV-1_pig	55.16%
Iotaarterivirus pejah_KR139839_PBJV_primate	Kappaarterivirus wobum_JN116253_WPDV_possum	68.96%
Iotaarterivirus pejah_KR139839_PBJV_primate	Lambdaarterivirus afriporav_KP026921_APRAV_rat	57.29%
Iotaarterivirus pejah_KR139839_PBJV_primate	Muarterivirus afrigant_MF324848_OSV-1_shrew	63.82%
Iotaarterivirus pejah_KR139839_PBJV_primate	Muarterivirus afrigant_MF324849_OSV-2_shrew	63.82%
Iotaarterivirus pejah_KR139839_PBJV_primate	Muarterivirus afrigant_MG264317_OSV-3_shrew	63.31%
Iotaarterivirus pejah_KR139839_PBJV_primate	Nuarterivirus guemel_KY369969_RtClanAV_rat	53.03%
Iotaarterivirus pejah_KR139839_PBJV_primate	Thetaarterivirus kafuba_KT447550_KKCBV_primate	34.59%
Iotaarterivirus pejah_KR139839_PBJV_primate	Thetaarterivirus mikelba1_KM110938_MYBV-1_primate	37.34%

Iotaarterivirus pejah_KR139839_PBJV_primate	Unclassified_KP280007_RtDs-Arterivirus -4_rat	53.03%
Iotaarterivirus pejah_KR139839_PBJV_primate	Unclassified_MT085132_RtRe-ArtV-Tka2010B_rat	58.59%
Iotaarterivirus pejah_KR139839_PBJV_primate	Unclassified_MT085133_RtRe-ArtV-Tka2010C_rat	58.33%
Iotaarterivirus pejah_KR139839_PBJV_primate	Unclassified_MT085136_RtRe-ArtV-Tl2006A_rat	58.08%
Iotaarterivirus pejah_KR139839_PBJV_primate	Unclassified_OQ715736_JSV-1_shrew	60.72%
Iotaarterivirus pejah_KR139839_PBJV_primate	Zetaarterivirus ugarco_KC787630_KRCV-1_primate	46.12%
Kappaarterivirus wobum_JN116253_WPDV_possum	Betaarterivirus europensis__KX668221_PRRSV-1_pig	69.72%
Kappaarterivirus wobum_JN116253_WPDV_possum	Betaarterivirus europensis_MT008024_PRRSV-1_pig	68.96%
Kappaarterivirus wobum_JN116253_WPDV_possum	Betaarterivirus europensis_MT311646_PRRSV-1_pig	69.21%
Kappaarterivirus wobum_JN116253_WPDV_possum	Lambdaarterivirus afriporav_KP026921_APRAV_rat	68.27%
Kappaarterivirus wobum_JN116253_WPDV_possum	Muarterivirus afrigant_MF324848_OSV-1_shrew	66.32%
Kappaarterivirus wobum_JN116253_WPDV_possum	Muarterivirus afrigant_MF324849_OSV-2_shrew	66.06%
Kappaarterivirus wobum_JN116253_WPDV_possum	Muarterivirus afrigant_MG264317_OSV-3_shrew	66.84%
Kappaarterivirus wobum_JN116253_WPDV_possum	Nuarterivirus guemel_KY369969_RtClanAV_rat	66.33%
Kappaarterivirus wobum_JN116253_WPDV_possum	Thetaarterivirus kafuba_KT447550_KKCBV_primate	68.37%
Kappaarterivirus wobum_JN116253_WPDV_possum	Thetaarterivirus mikelba1_KM110938_MYBV-1_primate	68.62%
Kappaarterivirus wobum_JN116253_WPDV_possum	Unclassified_KP280007_RtDs-Arterivirus -4_rat	66.33%
Kappaarterivirus wobum_JN116253_WPDV_possum	Unclassified_MT085132_RtRe-ArtV-Tka2010B_rat	68.37%
Kappaarterivirus wobum_JN116253_WPDV_possum	Unclassified_MT085133_RtRe-ArtV-Tka2010C_rat	68.37%
Kappaarterivirus wobum_JN116253_WPDV_possum	Unclassified_MT085136_RtRe-ArtV-Tl2006A_rat	68.62%
Kappaarterivirus wobum_JN116253_WPDV_possum	Unclassified_OQ715736_JSV-1_shrew	66.84%
Kappaarterivirus wobum_JN116253_WPDV_possum	Zetaarterivirus ugarco_KC787630_KRCV-1_primate	69.04%
Lambdaarterivirus afriporav_KP026921_APRAV_rat	Betaarterivirus europensis_MT008024_PRRSV-1_pig	58.04%
Lambdaarterivirus afriporav_KP026921_APRAV_rat	Betaarterivirus europensis_MT311646_PRRSV-1_pig	58.54%

Lambdaarterivirus afriporav_KP026921_APRAV_rat	Muarterivirus afrigant_MF324848_OSV-1_shrew	62.72%
Lambdaarterivirus afriporav_KP026921_APRAV_rat	Muarterivirus afrigant_MG264317_OSV-3_shrew	61.95%
Lambdaarterivirus afriporav_KP026921_APRAV_rat	Nuarterivirus guemel_KY369969_RtClanAV_rat	56.17%
Lambdaarterivirus afriporav_KP026921_APRAV_rat	Thetaarterivirus kafuba_KT447550_KKCBV_primate	59.95%
Lambdaarterivirus afriporav_KP026921_APRAV_rat	Thetaarterivirus mikelba1_KM110938_MYBV-1_primate	59.45%
Lambdaarterivirus afriporav_KP026921_APRAV_rat	Unclassified_MT085132_RtRe-ArtV-Tka2010B_rat	59.70%
Lambdaarterivirus afriporav_KP026921_APRAV_rat	Unclassified_MT085133_RtRe-ArtV-Tka2010C_rat	59.19%
Lambdaarterivirus afriporav_KP026921_APRAV_rat	Unclassified_MT085136_RtRe-ArtV-Tl2006A_rat	58.19%
Lambdaarterivirus afriporav_KP026921_APRAV_rat	Unclassified_OQ715736_JSV-1_shrew	59.13%
Lambdaarterivirus afriporav_KP026921_APRAV_rat	Zetaarterivirus ugarco_KC787630_KRCV-1_primate	57.89%
Muarterivirus afrigant_MF324848_OSV-1_shrew	Nuarterivirus guemel_KY369969_RtClanAV_rat	61.86%
Muarterivirus afrigant_MF324848_OSV-1_shrew	Thetaarterivirus kafuba_KT447550_KKCBV_primate	62.95%
Muarterivirus afrigant_MF324848_OSV-1_shrew	Thetaarterivirus mikelba1_KM110938_MYBV-1_primate	60.36%
Muarterivirus afrigant_MF324848_OSV-1_shrew	Unclassified_OQ715736_JSV-1_shrew	37.15%
Muarterivirus afrigant_MF324848_OSV-1_shrew	Zetaarterivirus ugarco_KC787630_KRCV-1_primate	61.08%
Muarterivirus afrigant_MF324849_OSV-2_shrew	Betaarterivirus europensis_MT008024_PRRSV-1_pig	60.57%
Muarterivirus afrigant_MF324849_OSV-2_shrew	Betaarterivirus europensis_MT311646_PRRSV-1_pig	60.82%
Muarterivirus afrigant_MF324849_OSV-2_shrew	Lambdaarterivirus afriporav_KP026921_APRAV_rat	62.21%
Muarterivirus afrigant_MF324849_OSV-2_shrew	Muarterivirus afrigant_MF324848_OSV-1_shrew	3.82%
Muarterivirus afrigant_MF324849_OSV-2_shrew	Muarterivirus afrigant_MG264317_OSV-3_shrew	4.58%
Muarterivirus afrigant_MF324849_OSV-2_shrew	Nuarterivirus guemel_KY369969_RtClanAV_rat	61.34%
Muarterivirus afrigant_MF324849_OSV-2_shrew	Thetaarterivirus kafuba_KT447550_KKCBV_primate	62.44%
Muarterivirus afrigant_MF324849_OSV-2_shrew	Thetaarterivirus mikelba1_KM110938_MYBV-1_primate	60.62%
Muarterivirus afrigant_MF324849_OSV-2_shrew	Unclassified_MT085132_RtRe-ArtV-Tka2010B_rat	59.02%

Muarterivirus afrigant_MF324849_OSV-2_shrew	Unclassified_MT085133_RtRe-ArtV-Tka2010C_rat	58.76%
Muarterivirus afrigant_MF324849_OSV-2_shrew	Unclassified_MT085136_RtRe-ArtV-Tl2006A_rat	58.76%
Muarterivirus afrigant_MF324849_OSV-2_shrew	Unclassified_OQ715736_JSV-1_shrew	36.90%
Muarterivirus afrigant_MF324849_OSV-2_shrew	Zetaarterivirus ugarco_KC787630_KRCV-1_primate	60.31%
Muarterivirus afrigant_MG264317_OSV-3_shrew	Betaarterivirus europensis_MT008024_PRRSV-1_pig	59.79%
Muarterivirus afrigant_MG264317_OSV-3_shrew	Betaarterivirus europensis_MT311646_PRRSV-1_pig	60.31%
Muarterivirus afrigant_MG264317_OSV-3_shrew	Muarterivirus afrigant_MF324848_OSV-1_shrew	5.09%
Muarterivirus afrigant_MG264317_OSV-3_shrew	Nuarterivirus guemel_KY369969_RtClanAV_rat	60.82%
Muarterivirus afrigant_MG264317_OSV-3_shrew	Thetaarterivirus kafuba_KT447550_KKCBV_primate	62.69%
Muarterivirus afrigant_MG264317_OSV-3_shrew	Thetaarterivirus mikelba1_KM110938_MYBV-1_primate	61.14%
Muarterivirus afrigant_MG264317_OSV-3_shrew	Unclassified_MT085132_RtRe-ArtV-Tka2010B_rat	59.79%
Muarterivirus afrigant_MG264317_OSV-3_shrew	Unclassified_MT085133_RtRe-ArtV-Tka2010C_rat	59.54%
Muarterivirus afrigant_MG264317_OSV-3_shrew	Unclassified_MT085136_RtRe-ArtV-Tl2006A_rat	59.02%
Muarterivirus afrigant_MG264317_OSV-3_shrew	Unclassified_OQ715736_JSV-1_shrew	36.39%
Muarterivirus afrigant_MG264317_OSV-3_shrew	Zetaarterivirus ugarco_KC787630_KRCV-1_primate	60.05%
Nuarterivirus guemel_KY369969_RtClanAV_rat	Thetaarterivirus kafuba_KT447550_KKCBV_primate	52.66%
Nuarterivirus guemel_KY369969_RtClanAV_rat	Thetaarterivirus mikelba1_KM110938_MYBV-1_primate	53.92%
Nuarterivirus guemel_KY369969_RtClanAV_rat	Unclassified_OQ715736_JSV-1_shrew	60.05%
Nuarterivirus guemel_KY369969_RtClanAV_rat	Zetaarterivirus ugarco_KC787630_KRCV-1_primate	53.54%
PP947442_ARTV/shrew/FS_shrew	Betaarterivirus americense_AY150564_PRRSV-2_pig	59.90%
PP947442_ARTV/shrew/FS_shrew	Betaarterivirus americense_EF112445_PRRSV-2_pig	59.38%
PP947442_ARTV/shrew/FS_shrew	Betaarterivirus americense_JN654459_PRRSV-2__pig	59.38%
PP947442_ARTV/shrew/FS_shrew	Betaarterivirus americense_JQ308798_PRRSV-2_pig	59.11%
PP947442_ARTV/shrew/FS_shrew	Betaarterivirus americense_U87392_PRRSV-2_pig	59.90%

PP947442_ARTV/shrew/FS_shrew	Betaarterivirus chinrav_KP280006_RtMrufAV_rat	57.81%
PP947442_ARTV/shrew/FS_shrew	Betaarterivirus europensis__KX668221_PRRSV-1_pig	58.85%
PP947442_ARTV/shrew/FS_shrew	Betaarterivirus europensis_JF802085_PRRSV-1_pig	59.01%
PP947442_ARTV/shrew/FS_shrew	Betaarterivirus europensis_M96262_PRRSV-1_pig	59.38%
PP947442_ARTV/shrew/FS_shrew	Betaarterivirus europensis_MT008024_PRRSV-1_pig	58.33%
PP947442_ARTV/shrew/FS_shrew	Betaarterivirus europensis_MT311646_PRRSV-1_pig	59.38%
PP947442_ARTV/shrew/FS_shrew	Betaarterivirus ninrav_KU302440_RtClonAV_rat	58.59%
PP947442_ARTV/shrew/FS_shrew	Betaarterivirus sheoin_KY369968_RtEiAV_rat	57.81%
PP947442_ARTV/shrew/FS_shrew	Betaarterivirus timiclar_KY369967_RtMcAV_rat	58.59%
PP947442_ARTV/shrew/FS_shrew	Deltaarterivirus hemfev_AF180391_SHFV_primate	57.70%
PP947442_ARTV/shrew/FS_shrew	Epsilonarterivirus hemcep_KM677927_SHEV_primate	59.38%
PP947442_ARTV/shrew/FS_shrew	Epsilonarterivirus safriver_KR862307_FSVV_primate	59.90%
PP947442_ARTV/shrew/FS_shrew	Epsilonarterivirus zamalb_KT166441_ZMbV-1_primate	58.85%
PP947442_ARTV/shrew/FS_shrew	Etaarterivirus ugarco_KC787658_KRCV-2_primate	60.31%
PP947442_ARTV/shrew/FS_shrew	Gammaarterivirus lacdeh_U15146_LDV_rat	58.07%
PP947442_ARTV/shrew/FS_shrew	Iotaarterivirus debrazmo_KP126831_DeBMAV_primate	57.44%
PP947442_ARTV/shrew/FS_shrew	Iotaarterivirus kibreg1_JX473849_KRTGV-1_primate	61.10%
PP947442_ARTV/shrew/FS_shrew	Iotaarterivirus pejah_KR139839_PBJV_primate	60.84%
PP947442_ARTV/shrew/FS_shrew	Kappaarterivirus wobum_JN116253_WPDV_possum	66.58%
PP947442_ARTV/shrew/FS_shrew	Lambdaarterivirus afriporav_KP026921_APRAV_rat	61.20%
PP947442_ARTV/shrew/FS_shrew	Muarterivirus afrigant_MF324848_OSV-1_shrew	44.30%
PP947442_ARTV/shrew/FS_shrew	Muarterivirus afrigant_MF324849_OSV-2_shrew	44.04%
PP947442_ARTV/shrew/FS_shrew	Muarterivirus afrigant_MG264317_OSV-3_shrew	44.30%
PP947442_ARTV/shrew/FS_shrew	Nuarterivirus guemel_KY369969_RtClanAV_rat	59.11%

PP947442_ARTV/shrew/FS_shrew	Thetaarterivirus kafuba_KT447550_KKCBV_primate	61.26%
PP947442_ARTV/shrew/FS_shrew	Thetaarterivirus mikelba1_KM110938_MYBV-1_primate	58.64%
PP947442_ARTV/shrew/FS_shrew	Unclassified_KP280007_RtDs-Arterivirus -4_rat	58.07%
PP947442_ARTV/shrew/FS_shrew	Unclassified_MT085132_RtRe-ArtV-Tka2010B_rat	57.29%
PP947442_ARTV/shrew/FS_shrew	Unclassified_MT085133_RtRe-ArtV-Tka2010C_rat	57.81%
PP947442_ARTV/shrew/FS_shrew	Unclassified_MT085136_RtRe-ArtV-Tl2006A_rat	57.81%
PP947442_ARTV/shrew/FS_shrew	Unclassified_OQ715736_JSV-1_shrew	46.11%
PP947442_ARTV/shrew/FS_shrew	Zetaarterivirus ugarco_KC787630_KRCV-1_primate	58.59%
PP964217_ARTV/rat/GD_rat	Betaarterivirus americense_AY150564_PRRSV-2_pig	48.74%
PP964217_ARTV/rat/GD_rat	Betaarterivirus americense_EF112445_PRRSV-2_pig	47.99%
PP964217_ARTV/rat/GD_rat	Betaarterivirus americense_JN654459_PRRSV-2__pig	47.74%
PP964217_ARTV/rat/GD_rat	Betaarterivirus americense_JQ308798_PRRSV-2_pig	48.24%
PP964217_ARTV/rat/GD_rat	Betaarterivirus americense_U87392_PRRSV-2_pig	48.74%
PP964217_ARTV/rat/GD_rat	Betaarterivirus chinrav_KP280006_RtMrufAV_rat	48.24%
PP964217_ARTV/rat/GD_rat	Betaarterivirus europensis__KX668221_PRRSV-1_pig	46.23%
PP964217_ARTV/rat/GD_rat	Betaarterivirus europensis_JF802085_PRRSV-1_pig	46.60%
PP964217_ARTV/rat/GD_rat	Betaarterivirus europensis_M96262_PRRSV-1_pig	46.98%
PP964217_ARTV/rat/GD_rat	Betaarterivirus europensis_MT008024_PRRSV-1_pig	46.48%
PP964217_ARTV/rat/GD_rat	Betaarterivirus europensis_MT311646_PRRSV-1_pig	47.24%
PP964217_ARTV/rat/GD_rat	Betaarterivirus ninrav_KU302440_RtClonAV_rat	44.72%
PP964217_ARTV/rat/GD_rat	Betaarterivirus sheoin_KY369968_RtEiAV_rat	46.98%
PP964217_ARTV/rat/GD_rat	Betaarterivirus timiclar_KY369967_RtMcAV_rat	46.48%
PP964217_ARTV/rat/GD_rat	Deltaarterivirus hemfev_AF180391_SHFV_primate	57.07%
PP964217_ARTV/rat/GD_rat	Epsilonarterivirus hemcep_KM677927_SHEV_primate	55.05%

PP964217_ARTV/rat/GD_rat	Epsilonarterivirus safriver_KR862307_FSVV_primate	54.55%
PP964217_ARTV/rat/GD_rat	Epsilonarterivirus zamalb_KT166441_ZMbV-1_primate	54.29%
PP964217_ARTV/rat/GD_rat	Etaarterivirus ugarco_KC787658_KRCV-2_primate	56.71%
PP964217_ARTV/rat/GD_rat	Gammaarterivirus lacdeh_U15146_LDV_rat	43.22%
PP964217_ARTV/rat/GD_rat	Iotaarterivirus debrazmo_KP126831_DeBMAV_primate	57.07%
PP964217_ARTV/rat/GD_rat	Iotaarterivirus kibreg1_JX473849_KRTGV-1_primate	54.80%
PP964217_ARTV/rat/GD_rat	Iotaarterivirus pejah_KR139839_PBJV_primate	58.33%
PP964217_ARTV/rat/GD_rat	Kappaarterivirus wobum_JN116253_WPDV_possum	66.33%
PP964217_ARTV/rat/GD_rat	Lambdaarterivirus afriporav_KP026921_APRAV_rat	60.20%
PP964217_ARTV/rat/GD_rat	Muarterivirus afrigant_MF324848_OSV-1_shrew	59.28%
PP964217_ARTV/rat/GD_rat	Muarterivirus afrigant_MF324849_OSV-2_shrew	59.02%
PP964217_ARTV/rat/GD_rat	Muarterivirus afrigant_MG264317_OSV-3_shrew	59.02%
PP964217_ARTV/rat/GD_rat	Nuarterivirus guemel_KY369969_RtClanAV_rat	46.48%
PP964217_ARTV/rat/GD_rat	PP947442_ARTV/shrew/FS_shrew	59.64%
PP964217_ARTV/rat/GD_rat	Thetaarterivirus kafuba_KT447550_KKCBV_primate	55.70%
PP964217_ARTV/rat/GD_rat	Thetaarterivirus mikelba1_KM110938_MYBV-1_primate	55.44%
PP964217_ARTV/rat/GD_rat	Unclassified_KP280007_RtDs-Arterivirus -4_rat	47.74%
PP964217_ARTV/rat/GD_rat	Unclassified_MT085132_RtRe-ArtV-Tka2010B_rat	24.37%
PP964217_ARTV/rat/GD_rat	Unclassified_MT085133_RtRe-ArtV-Tka2010C_rat	23.87%
PP964217_ARTV/rat/GD_rat	Unclassified_MT085136_RtRe-ArtV-Tl2006A_rat	25.13%
PP964217_ARTV/rat/GD_rat	Unclassified_OQ715736_JSV-1_shrew	59.79%
PP964217_ARTV/rat/GD_rat	Zetaarterivirus ugarco_KC787630_KRCV-1_primate	57.58%
Thetaarterivirus kafuba_KT447550_KKCBV_primate	Thetaarterivirus mikelba1_KM110938_MYBV-1_primate	25.81%
Thetaarterivirus kafuba_KT447550_KKCBV_primate	Zetaarterivirus ugarco_KC787630_KRCV-1_primate	46.37%

Thetaarterivirus mikelba1_KM110938_MYBV-1_primate	Zetaarterivirus ugarco_KC787630_KRCV-1_primate	45.36%
Unclassified_KP280007_RtDs-Arterivirus -4_rat	Betaarterivirus europensis__KX668221_PRRSV-1_pig	41.46%
Unclassified_KP280007_RtDs-Arterivirus -4_rat	Betaarterivirus europensis_MT008024_PRRSV-1_pig	41.46%
Unclassified_KP280007_RtDs-Arterivirus -4_rat	Betaarterivirus europensis_MT311646_PRRSV-1_pig	40.70%
Unclassified_KP280007_RtDs-Arterivirus -4_rat	Lambdaarterivirus afriporav_KP026921_APRAV_rat	54.91%
Unclassified_KP280007_RtDs-Arterivirus -4_rat	Muarterivirus afrigant_MF324848_OSV-1_shrew	57.99%
Unclassified_KP280007_RtDs-Arterivirus -4_rat	Muarterivirus afrigant_MF324849_OSV-2_shrew	57.99%
Unclassified_KP280007_RtDs-Arterivirus -4_rat	Muarterivirus afrigant_MG264317_OSV-3_shrew	57.47%
Unclassified_KP280007_RtDs-Arterivirus -4_rat	Nuarterivirus guemel_KY369969_RtClanAV_rat	38.94%
Unclassified_KP280007_RtDs-Arterivirus -4_rat	Thetaarterivirus kafuba_KT447550_KKCBV_primate	54.18%
Unclassified_KP280007_RtDs-Arterivirus -4_rat	Thetaarterivirus mikelba1_KM110938_MYBV-1_primate	52.41%
Unclassified_KP280007_RtDs-Arterivirus -4_rat	Unclassified_MT085132_RtRe-ArtV-Tka2010B_rat	47.24%
Unclassified_KP280007_RtDs-Arterivirus -4_rat	Unclassified_MT085133_RtRe-ArtV-Tka2010C_rat	46.73%
Unclassified_KP280007_RtDs-Arterivirus -4_rat	Unclassified_MT085136_RtRe-ArtV-Tl2006A_rat	47.24%
Unclassified_KP280007_RtDs-Arterivirus -4_rat	Unclassified_OQ715736_JSV-1_shrew	58.76%
Unclassified_KP280007_RtDs-Arterivirus -4_rat	Zetaarterivirus ugarco_KC787630_KRCV-1_primate	52.27%
Unclassified_MT085132_RtRe-ArtV-Tka2010B_rat	Betaarterivirus europensis_MT311646_PRRSV-1_pig	47.49%
Unclassified_MT085132_RtRe-ArtV-Tka2010B_rat	Muarterivirus afrigant_MF324848_OSV-1_shrew	59.28%
Unclassified_MT085132_RtRe-ArtV-Tka2010B_rat	Nuarterivirus guemel_KY369969_RtClanAV_rat	47.24%
Unclassified_MT085132_RtRe-ArtV-Tka2010B_rat	Thetaarterivirus kafuba_KT447550_KKCBV_primate	57.47%
Unclassified_MT085132_RtRe-ArtV-Tka2010B_rat	Thetaarterivirus mikelba1_KM110938_MYBV-1_primate	56.96%
Unclassified_MT085132_RtRe-ArtV-Tka2010B_rat	Unclassified_MT085133_RtRe-ArtV-Tka2010C_rat	2.76%
Unclassified_MT085132_RtRe-ArtV-Tka2010B_rat	Unclassified_MT085136_RtRe-ArtV-Tl2006A_rat	6.03%
Unclassified_MT085132_RtRe-ArtV-Tka2010B_rat	Unclassified_OQ715736_JSV-1_shrew	59.28%

Unclassified_MT085132_RtRe-ArtV-Tka2010B_rat	Zetaarterivirus ugarco_KC787630_KRCV-1_primate	56.57%
Unclassified_MT085133_RtRe-ArtV-Tka2010C_rat	Betaarterivirus europensis_MT311646_PRRSV-1_pig	47.74%
Unclassified_MT085133_RtRe-ArtV-Tka2010C_rat	Muarterivirus afrigant_MF324848_OSV-1_shrew	58.76%
Unclassified_MT085133_RtRe-ArtV-Tka2010C_rat	Nuarterivirus guemel_KY369969_RtClanAV_rat	47.24%
Unclassified_MT085133_RtRe-ArtV-Tka2010C_rat	Thetaarterivirus kafuba_KT447550_KKCBV_primate	57.22%
Unclassified_MT085133_RtRe-ArtV-Tka2010C_rat	Thetaarterivirus mikelba1_KM110938_MYBV-1_primate	56.71%
Unclassified_MT085133_RtRe-ArtV-Tka2010C_rat	Unclassified_MT085136_RtRe-ArtV-Tl2006A_rat	6.53%
Unclassified_MT085133_RtRe-ArtV-Tka2010C_rat	Unclassified_OQ715736_JSV-1_shrew	59.02%
Unclassified_MT085133_RtRe-ArtV-Tka2010C_rat	Zetaarterivirus ugarco_KC787630_KRCV-1_primate	56.57%
Unclassified_MT085136_RtRe-ArtV-Tl2006A_rat	Betaarterivirus europensis_MT311646_PRRSV-1_pig	48.24%
Unclassified_MT085136_RtRe-ArtV-Tl2006A_rat	Muarterivirus afrigant_MF324848_OSV-1_shrew	58.76%
Unclassified_MT085136_RtRe-ArtV-Tl2006A_rat	Nuarterivirus guemel_KY369969_RtClanAV_rat	45.98%
Unclassified_MT085136_RtRe-ArtV-Tl2006A_rat	Thetaarterivirus kafuba_KT447550_KKCBV_primate	57.72%
Unclassified_MT085136_RtRe-ArtV-Tl2006A_rat	Thetaarterivirus mikelba1_KM110938_MYBV-1_primate	56.46%
Unclassified_MT085136_RtRe-ArtV-Tl2006A_rat	Unclassified_OQ715736_JSV-1_shrew	58.25%
Unclassified_MT085136_RtRe-ArtV-Tl2006A_rat	Zetaarterivirus ugarco_KC787630_KRCV-1_primate	57.07%
Unclassified_OQ715736_JSV-1_shrew	Thetaarterivirus kafuba_KT447550_KKCBV_primate	61.14%
Unclassified_OQ715736_JSV-1_shrew	Thetaarterivirus mikelba1_KM110938_MYBV-1_primate	60.36%
Unclassified_OQ715736_JSV-1_shrew	Zetaarterivirus ugarco_KC787630_KRCV-1_primate	61.08%
